# Small Molecule IL-36γ Antagonist as a Novel Therapeutic Approach for Plaque Psoriasis

**DOI:** 10.1038/s41598-019-45626-w

**Published:** 2019-06-24

**Authors:** Viktor Todorović, Zhi Su, C. Brent Putman, Stevan J. Kakavas, Katherine M. Salte, Heath A. McDonald, Joseph B. Wetter, Stephanie E. Paulsboe, Qi Sun, Clare E. Gerstein, Limary Medina, Bernhard Sielaff, Ramkrishna Sadhukhan, Henning Stockmann, Paul L. Richardson, Wei Qiu, Maria A. Argiriadi, Rodger F. Henry, J. Martin Herold, J. Brad Shotwell, Steve P. McGaraughty, Prisca Honore, Sujatha M. Gopalakrishnan, Chaohong C. Sun, Victoria E. Scott

**Affiliations:** 1 AbbVie Inc., 1 North Waukegan Rd., North Chicago, IL 60064 USA; 2 AbbVie Bioresearch Center, 381 Plantation St., Worcester, MA 01605 USA

**Keywords:** Psoriasis, Drug discovery, Interleukins

## Abstract

IL-36 cytokines are pro-inflammatory members of the IL-1 family that are upregulated in inflammatory disorders. Specifically, IL-36γ is highly expressed in active psoriatic lesions and can drive pro-inflammatory processes in 3D human skin equivalents supporting a role for this target in skin inflammation. Small molecule antagonists of interleukins have been historically challenging to generate. Nevertheless, we performed a small molecule high-throughput screen to identify IL-36 antagonists using a novel TR-FRET binding assay. Several compounds, including 2-oxypyrimidine containing structural analogs of the marketed endothelin receptor A antagonist Ambrisentan, were identified as hits from the screen. A-552 was identified as a the most potent antagonist of human IL-36γ, but not the closely related family member IL-36α, was capable of attenuating IL-36γ induced responses in mouse and human disease models. Additionally, x-ray crystallography studies identified key amino acid residues in the binding pocket present in human IL-36γ that are absent in human IL-36α. A-552 represents a first-in-class small molecule antagonist of IL-36 signaling that could be used as a chemical tool to further investigate the role of this pathway in inflammatory skin diseases such as psoriasis.

## Introduction

Plaque psoriasis or psoriasis vulgaris (PV) is a chronic inflammatory skin disease whose initiation is not well understood but is mechanistically driven by the IL-17/IL-23 axis leading to keratinocyte hyper proliferation and resultant acanthosis, formation of characteristic rete ridges in the epidermis and significant immune cell infiltration into the dermis^1,2^. Current therapies for psoriasis include the use of targeted biologics such as anti-TNFα, anti-IL-17A and anti-IL-23 antibodies for moderate to severe patients^3^. The identification of orally active small molecule therapies for treating psoriasis is currently an area of active investigation. Efforts to modulate these cytokine pathways directly have been thwarted by the challenges associated with targeting disruption of protein-protein interactions between cytokines and their cognate receptors.

IL-36 family cytokines, which are members of the larger IL-1R superfamily, include three agonists (IL-36α, IL-36β, and IL-36γ) and an antagonist, IL-36Ra. All three agonists bind to and signal through a heterodimeric receptor composed of IL-36 receptor subunit (IL-36R), which binds selectively to all IL-36 family members and an IL-1RAcP co-receptor subunit, which is shared with several other IL-1R superfamily members including IL-1β. Binding of IL-36 agonists to IL-36R/IL-1RAcP heterodimer induces signaling through MyD88/IRAK complex leading to the activation of NF-κB and MAPKs signaling cascades shared among IL-1R superfamily members^4,5^.

IL-36 cytokines and their receptors are normally expressed at low levels but are significantly induced during inflammation in several tissues, including skin, joints, lung and gut^6,7^. Based on clinical data IL-36 cytokines may have a role in psoriasis as expression of IL-36γ is significantly upregulated in both serum and skin lesions of patients with PV and is normalized upon anti-TNFα treatment^8^. Moreover, loss of function mutations in the gene encoding IL-36Ra have been associated with generalized pustular psoriasis (GPP), a life threatening condition characterized by an increase in pro-inflammatory cytokines and immune cell infiltrate in skin^9^. Treatment of patients with GPP with an anti-IL-36R antibody has recently been reported to show robust efficacy^10,11^. The role of IL-36 in PV is less understood, however IL-36R knockout mice do not develop skin inflammation following topical application of the TLR7 agonist imiquimod (IMQ)^12^. In addition, overexpression of mouse IL-36α drives an inflammatory skin phenotype in mice^13^. Moreover, an antagonistic antibody to human IL-36R attenuated epidermal hyperplasia similar to etanercept in a human psoriasis xenograft model^14^. Recent studies have shown that chronic administration of mouse IL-36α into mouse ear skin leads to a thickening of the ear, epidermal hyperplasia, significant immune cell infiltration and dysregulation of key psoriasis relevant target genes that can be attenuated by pretreatment with an antagonistic mouse IL-36R antibody supportive of a direct role for IL-36 signaling in driving skin inflammation in mice^15^. Further assessment of mouse surrogate IL-36R antibodies in an IL-23 induced psoriasiform dermatitis mouse model showed significant attenuation of disease phenotype providing additional support for IL-36 driving disease in PV^15^. Given the chronic nature of skin inflammatory conditions such as PV, identification of small molecule antagonists of IL-36 signaling may provide a compelling alternative approach to biologic therapies. However, targeting cytokine ligand/receptor protein-protein interactions and subsequent signaling with small molecule modulators has proven challenging^16–18^. Recent advances in IL-1R structural biology, such as solving the structure of several IL-1R superfamily receptors and binding partners including IL-36R together with IL-36γ and IL-36Ra^19–23^, has opened the possibility for in-depth *in silico* analysis of potential small molecule binding sites and revealed the existence of such sites in IL-1R1^22,24,25^. Short peptide inhibitors of IL-1R have also been reported, demonstrating potential feasibility of such an approach^26,27^. Collectively, these findings support the possibility of targeting IL-36R/IL-36 agonist functional interaction sites with small molecule antagonists.

Herein, the discovery of small molecule IL-36γ antagonists was enabled through the implementation of a novel TR-FRET (Time-Resolved Fluorescence Resonance Energy Transfer) displacement assay whereby human IL-36γ binding to the extracellular domain of the human IL-36R/IL-1RAcP heterodimer was used to screen the AbbVie compound library. Subsequent triage of hits using cell-based IL-36γ/IL-1β evoked cytokine release assays identified biologically active small molecule antagonists of IL-36γ/IL-36R binding that abrogate receptor function with selectivity relative to IL-1β dependent signaling. Orthogonal biophysical assays confirmed direct IL-36γ binding of several series, including 2-oxypyrimidines exemplified by racemate A-706. Further characterization of the active enantiomer A-552 revealed interactions with key amino acid residues in human IL-36γ that are not present in mouse IL-36γ or human IL-36α. Furthermore, A-552 attenuated human IL-36γ induced pro-inflammatory responses in preclinical models. This is the first report of the identification of small molecule antagonists for the IL-36R/IL-36γ complex that are active in models of skin inflammation and provides the opportunity to target inhibition of this signaling pathway for treatment of multiple inflammatory indications.

## Methods

### Human skin

Human skin biopsies were acquired from three patients with psoriatic lesions in accordance with protocols approved by the Vista Medical Center East Institutional Review Board. Informed consent was obtained from all subjects, and the study was performed in adherence with the Declaration of Helsinki Principles. Biopsies (4 mm punch) of active psoriatic lesions as well as adjacent non-lesional skin were rinsed in PBS, placed in HypoThermosol FRS tissue transport solution (BioLife Solutions, Bothell, WA) with 200 μg/mL Penicillin, 200 μg/mL Streptomycin and 10 μg/mL Amphotericin for site-to-site transfer, moved to 10% NBF within 2 to 4 h of biopsy collection and fixed ≥36 h in 10% neutral buffered formalin between 2 foam biopsy pads in a tissue cassette.

### Histology, immunohistochemistry, *in situ* hybridization and imaging

A razor blade was used to halve samples for formalin-fixed, paraffin-embedded (FFPE) tissue processing (Leica) and embedding. Then 4 µm microtome sections were collected on slides (Leica).

Hematoxylin & eosin (H&E) staining and immunohistochemistry (IHC) were performed using a Leica ST5010 autostainer or Leica Bond RX automated immunostainer. Standardized IHC protocols were used for anti-loricrin staining (Abcam AB85679). Stained tissue slides were digitized for image analysis using a Perkin Elmer P250 whole slide digital pathology slide scanner with 20X objective and extended focus scanning parameters. For quantitative analysis, N = 3 raft samples were analyzed per condition for each stain using whole slide digital analysis software (Indica Labs HALO). Staining was normalized to the length of the raft sample analyzed.

Automated chromogenic *in situ* hybridization (ISH) was conducted using an immunostainer (Bond RX; Leica), RNAscope® 2.5 LS Reagent Kit-RED (Advanced Cell Diagnostics) and Refine Red Detection kit (DS9390; Leica). Advanced Cell Diagnostic’s (ACD) standard automated ISH protocol was used after optimizing pre-treatment conditions for human skin (ER2 for 15 min at 88 °C). Probes used in this study were all designed by and ordered from ACD and included a positive control probe, PPIB; a negative control probe, DapB; test probes, IL-36α; IL-36γ; IL-36R and IL-36Ra. All stained tissue slides were digitized for image analysis using a whole slide digital pathology slide scanner with 40X objective and extended focus scanning parameters (P250; Perkin Elmer). Quantitative analysis was performed by manually drawing ROI’s around the epidermis (excluding ~200 µm of tissue edge) and by creating a 200 µm margin from the bottom of the basal epidermal layer to create the dermal ROI. RNA signal in the epidermis and dermis was counted using whole slide digital analysis software (HALO; Indica Labs) and normalized by tissue length. Statistical significance was determined using an unpaired, two-tailed t test comparing the normalized RNA signal counts in lesional samples to the normalized RNA signal counts in non-lesional samples for each of the probes.

### IL-36γ induced 3D skin equivalent model

3D skin equivalents (SOR-300-FT-CTR) comprised of human neonatal keratinocytes and fibroblasts were purchased from MatTek, equilibrated for one day in proprietary formulated media (SOR-300-FT-CTR-MM) and then treated as indicated. Treatment groups contained hIL-36γ at 300 ng/mL, 10 μM of A-552, 10 μg/mL anti-IL36R antibody, 10 μg/mL IL-36RA, or 10 μg/mL IgG isotype control either alone or in combination with hIL-36γ. Control skin equivalents were treated with media only. Skin equivalents were treated for three days, with media collected for cytokine release studies at day two and replaced with fresh treatment media. After three days the skin equivalents were either placed in 10% neutral buffered formalin solution (for histology) or frozen (for qRT-PCR).

### Cloning, expression and purification of proteins

Expression of His6-SUMO-human IL-36γ (18–169) and His6-SUMO-human IL-36Ra (2–155) was performed in *E*. *coli* strain BL21 (DE3)(Life Technologies) as previously described^28^. Initial purifications were performed by affinity chromatography on a nickel column, followed by cleavage of the His-SUMO tag by ULP1 protease. The cleaved proteins were further purified by gel filtration on a HiLoad 26/60 Superdex 75 column. Expression and purification of the hIL-36R/IL-1RAcP extracellular domain (ECD) knob-in-hole Fc was performed as previously described^22,28,29^. Briefly, baculovirus were generated for each component using the Bac-to-Bac method (Life Technologies), and then co-infected into Sf9 cells (Life Technologies). After 72 h of growth at 27 °C the cells were removed by centrifugation and the culture supernatant containing the secreted protein was filtered through a Pall AcroPak 500 filtration device. Initial purifications were performed by affinity chromatography on a nickel column followed by dialysis into PBS. The eluted material was mixed with 10 mL of a hIL-36α conjugated affinity resin. The affinity resin was prepared using biotinylated hIL-36α protein that was bound to streptavidin agarose beads (Thermo Fisher Scientific). After overnight incubation at 4 °C, the bound resin was washed with PBS, and the heterodimer was eluted by the addition of 0.1 M glycine, pH 3.7, which was immediately neutralized by adding 1/10 volume of 0.5 M Tris, pH 8.0, 1.5 M NaCl. Fractions containing the IL-36R/IL-1RAcP heterodimer were pooled, dialyzed into PBS, pH 7.4, and concentrated to the desired concentration.

### Protein labeling

Purified IL-36γ was labeled using Alexa Fluor 488 N-hydroxysuccinimidyl ester (NHS) and Alexa Fluor 647 NHS (Molecular Probes) according to the manufacturers’ protocol. Successful labeling of the ligands was confirmed using mass spectrometry. Impurities and excess dyes were removed, and conjugated proteins were transferred to 1X PBS pH 7.4 (Sigma) using Zeba Spin 7K desalting columns (Thermo Scientific).

### TR-FRET assays

TR-FRET assays were performed in low-volume 384-well Proxy Plates (Perkin Elmer). 5 μL of assay buffer (1% BSA in 1 X PBS), 5 μL of either human IL-36R/IL-1RAcP ECD knob-in-hole Fc or human IL-36R His tagged protein (1.5 μg/mL final concentration) in assay buffer and 5 μL of detection solution (6 nM final concentration of LanthaScreen Elite Tb-anti-His Antibody (Thermo Scientific) together with either NHS-AlexaFluor-488 conjugated IL-36 cytokines at concentrations indicated) were added to individual wells. Plates were sealed and incubated overnight at 4 °C. After the incubation, plates were re-equilibrated to room temperature and the ratio between fluorescent signals at 520 nm and 495 nm (TR-FRET ratio) was detected using the TR-FRET enabled EnVision plate reader (Perkin Elmer). To perform the TR-FRET assay in competitive mode, 5 μL of unlabeled human IL-36γ or human IL-36Ra was added at indicated concentrations, 5 μL of human IL-36R/IL-1RAcP ECD knob-in-hole Fc (1.5 μg/mL final concentration) in assay buffer and 5 μL of detection solution (6 nM final concentration of LanthaScreen Elite Tb-anti-His Antibody (Thermo Scientific) together with either NHS-AlexaFluor-488 conjugated human IL-36γ at 6 nM final concentration). EC_50_/IC_50_ values were estimated from a four-parameter logistic fit using Prism software (GraphPad). Ki values of antagonists were calculated using Cheng-Prusoff equation $Ki=\frac{IC50}{1+A\,/\,EC50}$ (IC_50_ of the antagonist, A agonist concentration, EC_50_ of the agonist). All values were presented as mean ± standard deviation (S. D.).

To adapt the TR-FRET assay for high throughput screening the protocol was adapted as follows. The Echo liquid handler was used to transfer 30 nL per well of 5 mM stock compounds in DMSO to white Proxy Plates, that were foil sealed and stored at room temperature until use. On the day of an assay, the Multi-Drop Combi was used to dispense 2.5 μl of 2X receptor (human IL-36R/IL-1RAcP ECD knob-in-hole Fc) onto these compound plates, followed by one-hour incubation. Next, 2.5 μL of a 2X detection mix consisting of either Tb-Anti-His antibody with human IL-36γ-Alexa488 or Eu-Anti-His antibody with human IL-36γ-Alexa647 was added to bring the final assay volume to 5.0 μL and the compound concentration to 30 μM. After an additional 4 h of incubation, the plates were read in the Envision as previously described.

### IL-6, IL-8 and CXCL1 release assays

The immortalized human keratinocyte cell line HaCaT and mouse fibroblast NIH-3T3 cell line were maintained in culture in DMEM with 10% fetal bovine serum and 1% penicillin/streptomycin added (Sigma). Cells were seeded in 96-well tissue culture plates (Corning) at 25,000 cells per well and allowed to adhere and recover overnight at 37 °C in an incubator. Cells were rinsed with PBS (Sigma) and treated with cytokines diluted in 200 μL/well of the medium (DMEM with 0.5% FBS and 1% penicillin/streptomycin) as indicated. In inhibition mode, the agonist concentration used was at the EC_80_ for chemokine production and the cells were pre-treated with inhibitors for 30 min prior to adding the agonist. After 24 h of treatment, supernatants were collected and IL-8 and CXCL1^28^ levels were assessed using a custom immunoassay (Meso Scale Discovery). EC_50_/IC_50_ values were estimated from a four-parameter logistic fit using Prism software (GraphPad).

The IL-8 release assays were miniaturized for HTS hit follow-up. Frozen aliquots of HaCaT cells were thawed, added to complete media, mixed, spun down, resuspended in fresh media, and dispensed using the Multidrop384 into black, clear-bottom 384 well plates at a final density of 4000 cells/well. After a 24 h recovery period at 37 °C/5% CO_2_, the cell plates were rinsed twice with 40 μL/well fresh media. The Biomek FX workstation was then used to add 10 μL/well compounds. Cell plates were then returned to 37 °C for 15 min incubation, followed by the addition of 10 μl/well of IL-36γ (40 ng/ml final). After an additional 5 h of incubation at 37 °C, 5 μl/well of cell supernatant was removed with the Biomek FX and transferred to a 384 well Proxy Plate for IL-8 detection using the HTRF kit from Cisbio. For the IL-1β evoked release of IL-8, the same protocol was followed, but with a final concentration of 1 ng/mL IL-1β and an overnight incubation instead of 5 h. Cell viability was assessed using Cell Titer-Glo (Promega) according to the manufacturers’ instructions.

To measure IL-6, IL-8 and CXCL1 release from 3D skin equivalents diluted sample media collected at day 2 of the treatment (see above) was used in conjunction with V-PLEX multiplex immunoassay for IL-6 and IL-8 and a U-PLEX immunoassay for CXCL1 (Meso Scale Discovery) according to the manufacturers’ protocols.

### Tool compound synthesis

A-706 was present in the AbbVie high throughput screening collection and following the screen its purity and identity were confirmed by ^1^H NMR and LCMS. Subsequently individual enantiomers A-552 and A-553 were synthesized as described in the Supplementary Information.

### Thermal shift assay

Thermal shift assays^30^ were performed on a Roche LightCycler 480 instrument using Sypro Orange Dye (Invitrogen). Reactions consisted of 0.5–2 µM purified recombinant protein in PBS, pH 7.4, a 1/500 dilution of the stock Sypro Orange reagent, and 100 µM test compounds, resulting in a final DMSO concentration of 1%. The samples were heated from 20 °C to 95 °C at a rate of 0.25 °C per second.

### Isothermal titration calorimetry

All experiments were carried out on a MicroCal^TM^ Auto-ITC200 at 25 °C in 50 mM HEPES (pH = 7.5), 150 mM NaCl, 0.5 mM TCEP, 2% DMSO. IL-36γ protein solution was buffer exchanged by gel filtration into the isothermal titration calorimetry (ITC) buffer and diluted to 40 µM. The titrations were conducted using an initial injection of 0.2 µL followed by 19 identical injections of 2 µL of 400 µM compound solution. Thermodynamic parameters were calculated using ∆G = ∆H − T∆S = −RTlnKB, where ∆G, ∆H and ∆S are the changes in free energy, enthalpy and entropy of binding respectively. A single binding site model was employed.

### NMR studies

2D [^1^H,^13^C]-HSQC and 2D [^1^H,^15^N]-HSQC spectra were acquired on 50 µM protein samples uniformly ^15^N labeled and ^13^C labeled at the terminal methyl groups of leucine, valine, isoleucine and methionine in buffer containing 50 mM Tris pH 7.5, 100 mM NaCl, 0.5 mM TCEP, 90:10 H_2_O:D_2_O, with and without 250 µM of A-552. Data were recorded at 303 K on DRX500 spectrometers equipped with a cryoprobe (Bruker). Screening data were processed using Bruker TOPSPIN and analyzed by comparing spectra with and without compounds.

### X-ray Crystallography

IL-36γ: A-552 complex was prepared using low concentration complexing technique. Specifically, purified IL-36γ protein was first diluted to the concentration of 1 mg/mL using the purification buffer (150 mM NaCl, 0.5 mM TCEP, 50 mM HEPES pH7.5). A-552 was then added to the protein sample to a final concentration of 1 mM. The mixture was incubated at 37 °C for 2 h and then further concentrated to 20 mg/mL. The crystallization experiments were carried out by using sitting drop vapor diffusion method. 100 nL of IL-36γ and A-552 complex were mixed with 100 nL of crystallization solution (3 M NaCl, 0.1 M Na_3_ Citrate pH 3.5) and incubated at 23 °C. The crystals appeared after one day of incubation and grew to their full size after 3 days. Crystals were flash frozen in liquid nitrogen using 20% ethylene glycol plus crystallization solution as cryo-protectant. X-ray diffraction data were collected at IMCA-CAT beam line 17-ID at Argonne National Laboratory and processed to 2.0 Å resolution.

### Structure determination of human IL-36γ:A-552 complex

Diffraction data were processed using the program AUTOPROC from Global Phasing Ltd^31^. The dataset was processed in the space group I23 with the following unit cell dimensions: a = b = c = 96.4. A maximum likelihood molecular replacement solution was determined using the program PHASER^32^ using an IL-36γ search model reported previously (Protein Data Bank entry 4IZE^22^). Coordinates for one IL-36γ molecule were generated based on the molecular replacement solution. A-552 ligand dictionary was calculated using the program GRADE^33^ and ligand fitting into difference density was conducted using the program RHOFIT^34^. Preliminary refinement of the resulting solution was conducted using the program BUSTER^35^. Iterative protein model building was conducted using the program COOT^36^ and examination of 2Fo-Fc and Fo-Fc electron-density maps. Refinement concluded with the addition of water molecules using BUSTER. Final refinement statistics reported R_free_/R_work_ values of 0.26/0.22. All structural figures were created in the program Pymol (Schrӧdinger)^37^. Structural coordinates have been deposited to the Protein Data Bank (www.rcsb.org) with the following PDB code: 6P9E.

The ternary homology model of human IL-36γ/IL-36R/IL-1RAcP and homology models of human IL-36α and IL-36β and mouse IL-36γ were created using the program Prime from Schrödinger’s Small-Molecule Drug Discovery Suite of programs^38^.

### qRT-PCR

RNA from frozen 3D skin equivalents was isolated using miRNeasy Mini Kit (Qiagen) per the manufacturers’ instructions. For qRT-PCR determination a CFX384 Real-Time System C1000 Touch Thermocycler was used. Briefly, in a 384 well plate, 2 μL of sample RNA (10 ng/µL) was added to each well. 8 μL of TaqMan mix from TaqMan RNA-to-C_T_ 1-Step Kit (Applied Biosciences) and the appropriate primers were added to each well. Primers for RPLP0 (Hs99999902_m1), S100A7 (Hs01923188_u1), DEFB4B (Hs00175474_m1), elafin (Hs00160066_m1), keratin 16 (Hs00955088_g1), loricrin (Hs01894962_s1), involucrin (Hs00846307_s1), and keratin 10 (Hs01043114_g1) were purchased from ThermoFisher Scientific. Each transcript measured was presented as a fold change compared to control using the ∆∆C_T_ method as RNA fold increase where RPLP0 was used as a housekeeping gene.

### IL-36γ induced CXCL1 release in mouse plasma

Female C57BL/6 mice (9–10 weeks old, 19–23 g) were purchased from Charles River Laboratories. All animal procedures were approved by AbbVie’s Institutional Animal Care and Use Committee (IACUC) and performed in accordance with the relevant IACUC guidelines and regulations. To examine engagement of human IL-36γ with antagonists under *in vivo* conditions, a mouse acute CXCL1 release assay was established which was similar to one previously reported^15^. Plasma CXCL1 secretion was induced by subcutaneous injection of 2 μg human IL-36γ (in 100 μL PBS) into C57BL/6 mice. Blood was withdrawn via cardiac puncture 2 h after the challenge with IL-36γ. A-552 and A-553 were dosed at 10, 30 and 100 mg/kg p.o. 45 min before IL-36γ challenge. Anti-IL36R antibodies were dosed i.p. at 24 hours before IL-36γ challenge. Plasma CXCL1 levels were analyzed using MSD V-PLEX kit.

## Results

### IL-36γ expression was upregulated in psoriatic lesions and drove psoriasiform changes in 3D skin equivalents

**Figure 1:**
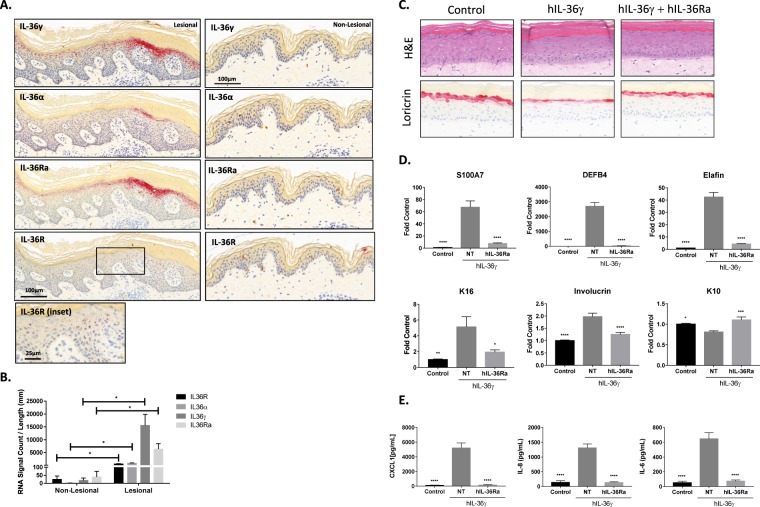
IL-36γ is upregulated in psoriatic lesions and drives psoriasiform changes in 3D skin equivalents. (**A**) *In situ* hybridization images of psoriasis patient tissue isolated from a lesion (left) or non-lesional skin (right). Red spots represent RNA signal for the select probes; images representative of three patients are shown. (**B**) Differences between lesional and non-lesional RNA signals were quantified for each probe represented in. (**A**) Average of 3 patients is shown ± S.E.M. (*p < 0.05 in an unpaired, two-tailed t test; df = 4, t = 4.458 (IL-36α), 3.633 (IL-36γ), 4.289 (IL-36R), 3.027 (IL-36Ra)). (**C**) H&E (top) and loricrin (bottom) staining of fully differentiated 3D skin equivalents untreated or treated with 0.3 μg/mL hIL-36γ with or without the presence of 10 μg/mL of function blocking hIL-36Ra. Representative images at 32X magnification are shown. (**D**) Differences between RNA transcripts in treated and non-treated 3D skin equivalents were quantified for each probe using qRT-PCR. Average of 6 treated and 7 control is shown ± S.E.M. (*p < 0.05, **p < 0.01, ***p < 0.001, ****p < 0.0001 using ANOVA with Dunnett’s post hoc test vs. hIL-36γ alone; (F; DF) = 38.58; 18 (S100A7), 114.3; 18 (DEFB4), 129.5; 18 (Elafin), 8.934; 18 (K16), 31.49; 18 (Involucrin), 10.96; 18 (K10)). (**E**) Differences between secreted CXCL1, IL-8 and IL-6 proteins in treated and non-treated 3D skin equivalents were quantified using immunoassays. Average of 6 treated (5 in CXCL1 and IL-8 NT column) and 7 control samples is shown ± S.E.M. (*p < 0.05, **p < 0.01, ***p < 0.001, ****p < 0.0001 using ANOVA with Dunnett’s post hoc test vs. hIL-36γ alone; (F, DF) = 70.64; 17 (CXCL1), 72.23; 17 (IL-8), 49.54; 18 (IL-6)).

To assess the relative expression of IL-36 cytokines in psoriasis skin, mRNA levels of each isoform were measured from patient biopsies by quantitative *in situ* hybridization (Fig. 1A). IL-36α, IL-36γ, IL-36Ra and IL-36R were all significantly elevated in the epidermis of lesional psoriatic human skin when compared to non-lesional (Fig. 1A,B). The results for all 3 donors were conclusive for expression patterns. The high levels of IL-36γ and IL-36Ra in the lesional samples were localized to the upper stratum spinosum and granulosum layers. Largest increases in IL-36γ and IL-36Ra were focused beneath areas of parakeratotic stratum corneum, an indication of disease activity in those regions of the plaque. The increase in IL-36α is mainly localized to the stratum granulosum layer and is modest in contrast to the more robust IL-36γ and IL-36Ra expression. IL-36R was ubiquitously expressed at a low level throughout all layers of the epidermis. The positive (PPIB) and negative (DapB) control probe slides showed expected staining patterns in these tissue sections (see Supplementary Fig. 1).

The 3D skin equivalent model system has often been used to assess the impact of various treatments, including IL-36, on keratinocyte differentiation and stratification^39–41^ and was used here to test the hypothesis that hIL-36γ can drive a psoriatic phenotype in human epidermis similar to studies recently reported for IL-36 family members^40,41^. Addition of hIL-36γ resulted in morphological changes consistent with psoriasis in the model with a prominent increase in thickness of the cornified layer (hyperkeratosis) and a concomitant decrease in the granular layer (hypogranulosis) as well as a significant decrease in the terminal differentiation marker loricrin (Fig. 1C; Supplementary Fig. 2). Moreover, IL-36γ induced a psoriasis-like gene expression pattern (Fig. 1D) as well as inducing the production of pro-inflammatory chemokines (Fig. 1E) related to the disease. Importantly, IL-36γ induced phenotypes were fully attenuated by recombinant soluble hIL-36Ra, further confirming the specificity of cytokine induced activity on the IL-36R (Fig. 1C–E).

### Development and validation of IL-36 binding and functional assays

Previously published reports showing direct protein-protein binding data determined using surface plasmon resonance (SPR) clearly demonstrated that IL-36α and γ bind to recombinant monomeric IL-36R ECD with low affinity and to the recombinant IL-36R/IL-1RAcP ECD heterodimer with high affinity (μM versus sub-nM range, respectively), whereas IL-36Ra potently binds to both monomer and heterodimer in the low nM range^22,28^. However, screening to identify small molecule inhibitors of IL-36 signaling would be challenging using SPR as this technique requires large amounts of high-quality stable protein. Towards this end, a novel TR-FRET^42^ assay was developed to enable high throughput screening of the AbbVie small molecule library of 850,000 compounds.

**Figure 2:**
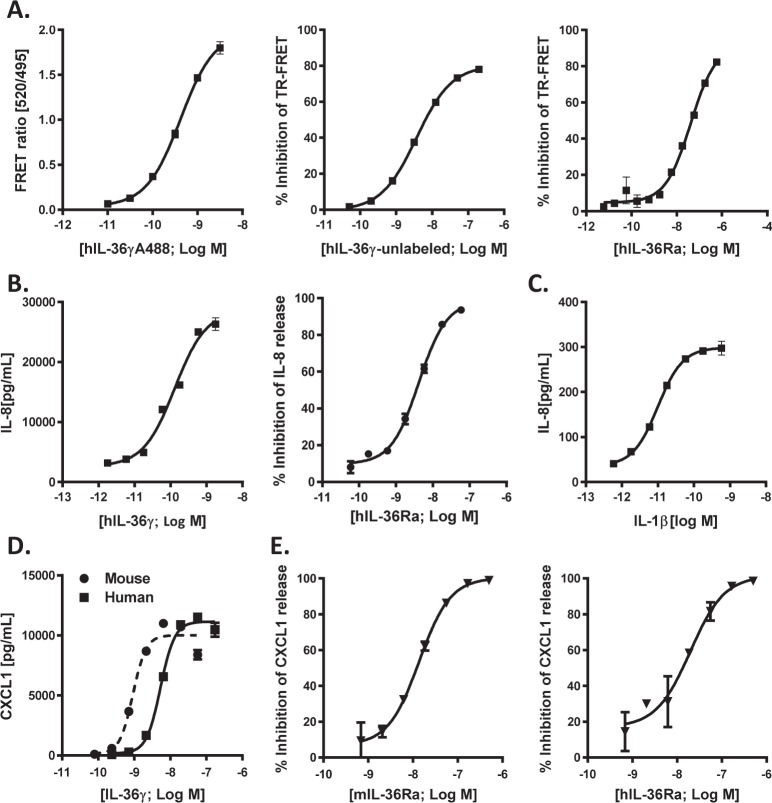
Validation of hIL-36γ binding and functional assays for compound triage. (**A**) Binding between Alexa-488 labeled hIL-36γ and hIL-36R/hIL-1RAcP ECD heterodimer was measured by FRET ratio (left panel, n = 3). Inhibition of interaction between labeled hIL-36γ and heterodimer was assessed by displacement using either unlabeled hIL-36γ (middle panel, n = 3) or hIL-36Ra (right panel, n = 2). (**B**) hIL-36γ function was measured by its ability to induce the secretion of IL-8 in human keratinocyte derived cell line HaCaT (left panel, n = 3). Ability to inhibit hIL-36γ function was assessed using hIL-36Ra (right panel, n = 3). (**C**) hIL-1β function was measured by its ability to induce the secretion of IL-8 in HaCaT cells, n = 3. (**D**) Human-to-mouse cross-species activity of hIL-36γ was measured by its ability to induce the secretion of CXCL1 from mouse NIH 3T3 cells, n = 2. (**E**) Specificity of cross-species activity of hIL-36γ was tested by functional inhibition using mouse (left panel, n = 2) and human (right panel, n = 2) IL-36Ra. All data represents an average ± S.E.M.

In the TR-FRET assay, N-terminal labeled Alexa 488 human IL-36γ produced a robust signal window binding to human IL-36R/IL-1RAcP ECD Fc heterodimer (Fig. 2A) which is in line with direct binding data reported for unlabeled IL-36γ^22,28^ (EC_50_ = 0.3 ± 0.1 nM, n = 7). Ligand/receptor binding specificity was validated using unlabeled IL-36γ and IL-36Ra, which both inhibit this cytokine binding interaction (Fig. 2A) as expected (Ki = 0.4 ± 0.2 nM, n = 9 and Ki = 2.0 ± 1.7 nM, n = 4, respectively). Since IL-36γ can induce a robust release of pro-inflammatory cytokines and chemokines from keratinocytes^43,44^ an IL-36γ evoked IL-8 release assay from HaCaT cells was established and validated by inhibition with IL-36Ra (Fig. 2B) with results consistent with the TR-FRET assay values (EC_50_ = 0.2 ± 0.1 nM, n = 4 and Ki = 0.6 ± 0.1 nM, n = 2, respectively). This assay was also used to confirm that the activity of Alexa 488 labeled IL-36γ is maintained similar to unlabeled cytokine demonstrating that the modified ligand retained its biological activity (Supplementary Fig. 3A). To triage compounds non-selectively hitting the IL-1RAcP, an IL-1β evoked IL-8 release assay was also established and validated (Fig. 2C) yielding the expected potency values (EC_50_ = 5.7 ± 3.8 pM, n = 3).

### Human and mouse IL-36γ showed asymmetric species cross-reactivity with cognate receptors

Previously, mouse IL-36α has been shown to induce the release of CXCL1 from mouse NIH-3T3 cells and mouse IL-36Ra can block this release^44^. Herein, these studies have been extended to show that mouse IL-36γ can also induce the release of CXCL1 from NIH-3T3 cells. Moreover, human IL-36γ can also induce the release of CXCL1 from NIH-3T3 cells albeit with a slightly lower potency (Fig. 2D) than mouse IL-36γ (EC_50_ = 0.6 ± 0.2 nM, n = 8 versus EC_50_ = 2.4 ± 1.8 nM, n = 9, respectively). Interestingly, mouse IL-36Ra and human IL-36Ra can similarly attenuate CXCL1 release induced by human IL-36γ (Fig. 2E) (IC_50_ = 19 ± 9 nM, n = 4 and IC_50_ = 23 ± 10 nM, n = 4, respectively). In contrast, mouse IL-36γ cannot induce CXCL1 release from HaCaT cells (Supplementary Fig. 3B) demonstrating asymmetric species cross-reactivity between mouse and human IL-36 cytokines and their cognate receptors.

### Small molecule antagonists of IL-36R signaling identified by High Throughput Screening

**Figure 3:**
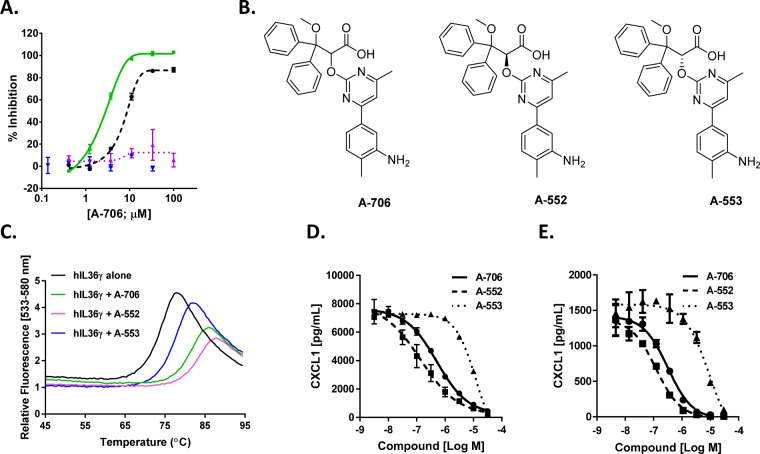
Identification and characterization of small molecule inhibitor of IL-36γ signaling. (**A**) Activity of the HTS hit A-706 in binding (TR-FRET, black), functional (IL-8 driven production, hIL-36γ (green) and IL-1β (blue)), and cell toxicity (purple) assays. (**B**) Depicts the chemical structure of the racemate A-706 and its two resolved enantiomers A-552 and A-553. (**C**) Thermal shift assay for hIL-36γ in the absence (black) and in the presence of 100 μM of A-706 (green), A-552 (purple) and A-553 (blue). **(D**–**E**) Potency of A-706, A-552 and A-553 was measured in human **(D)** and mouse **(E**) cells using hIL-36γ induced secretion of CXCL1. Data represents an average of duplicate experiments ± S.E.M.

The high throughput screen was performed as detailed in Supplementary Table 1. The TR-FRET assay was adapted for high throughput screening (see Online Methods) to screen the AbbVie small molecule compound library for compounds that could disrupt human IL-36γ ligand/receptor binding. A total of 542 hits showed robust dose-dependent inhibition of the interaction between human IL-36γ and the human IL-36R/IL-1RAcP ECD Fc heterodimer. Following identification, these hits were subsequently advanced into the human IL-36γ and human IL-1β evoked IL-8 release cell-based assays in addition to performing cell viability assays in HaCaT cells as a counter screen to assess selectivity. The top hit A-706 from the 2-oxypyrimidine series showed similar potency in the TR-FRET binding assay and the hIL-36γ induced IL-8 cell based functional assay but no activity in the hIL-1β induced IL-8 release assay or cellular toxicity (Fig. 3A) in HaCaT cells. Oxypyrimidine A-706 was resolved to its constituent enantiomers (Fig. 3B) which were further assessed individually for their affinity for human IL-36α or human IL-36γ in a thermal shift assay (TSA) using recombinant truncated proteins (Fig. 3C; Supplementary Fig. 4). A-706 increased the melting temperature of human IL-36γ by 5.4 °C at 100 μM demonstrating protein stabilization and confirming ligand binding. A-552 and A-553 increased the melting temperatures of human IL-36γ by 6.1 °C and 1.3 °C, respectively, in the IL-36γ TSA (Fig. 3C), indicating that A-552 binds the cytokine with higher affinity. The potency of A-552 and A-553 was also assessed in the human IL-36γ evoked CXCL1 release in both human (Fig. 3D) and mouse (Fig. 3E) cellular assays yielding K_i_ values shown in Table 1 indicating that A-552 is the more potent antagonist of IL-36γ activity in these functional assays.

**Table 1:** Compound potencies in the human IL-36γ evoked CXCL1 release assays from cells.

Compound	Human HaCaT cells, Ki	Mouse NIH-3T3 cells, Ki
A-706	121 ± 32 nM	181 ± 54 nM
A-552	31 ± 22 nM	54 ± 10 nM
A-553	3.7 ± 2.3 μM	3.7 ± 1.7 μM

Ki average 3 independent experiments (n = 4 Human HaCaT A-552 and A-553, n = 2 for Mouse NIH 3T3 A-553) is shown ± S.D.

### A-552 directly binds to human IL-36γ in biophysical assays

**Figure 4:**
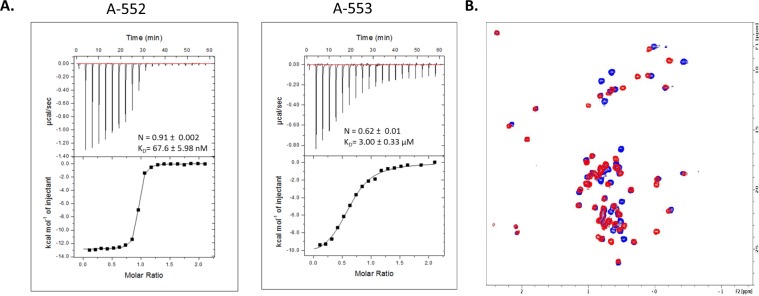
A-552 directly binds to human IL-36γ. **(A**) ITC curves for hIL-36γ direct binding to A-553 and A-553. (**B**) 2D [^1^H-^13^C] methyl HSQC spectra of hIL-36γ with (red) and without (blue) added A-552. Multiple chemical shift perturbations suggest robust binding.

Direct physical interaction between A-552 and A-553 with human IL-36γ was quantitatively measured by ITC. This analysis defined K_D_ values of 67.6 ± 6 nM and 3000 ± 330 nM for A-552 and A-553, respectively (Fig. 4A). These values are consistent with the K_i_ values determined for these two compounds in human IL-36γ evoked CXCL1 release from mouse and human cells (Fig. 3D,E). Labeling the human IL-36γ protein with ^13^C and ^15^N during the expression of recombinant protein in *E coli* enabled NMR analysis and robust binding observed with A-552 by NMR as evidenced by multiple chemical shift perturbations observed upon addition of the compound (Fig. 4B). The fact that the chemical shift perturbations were not localized to a small number of peaks suggested the potential conformational changes induced with compound binding.

### Crystal structure of A-552 bound to hIL-36γ

**Figure 5:**
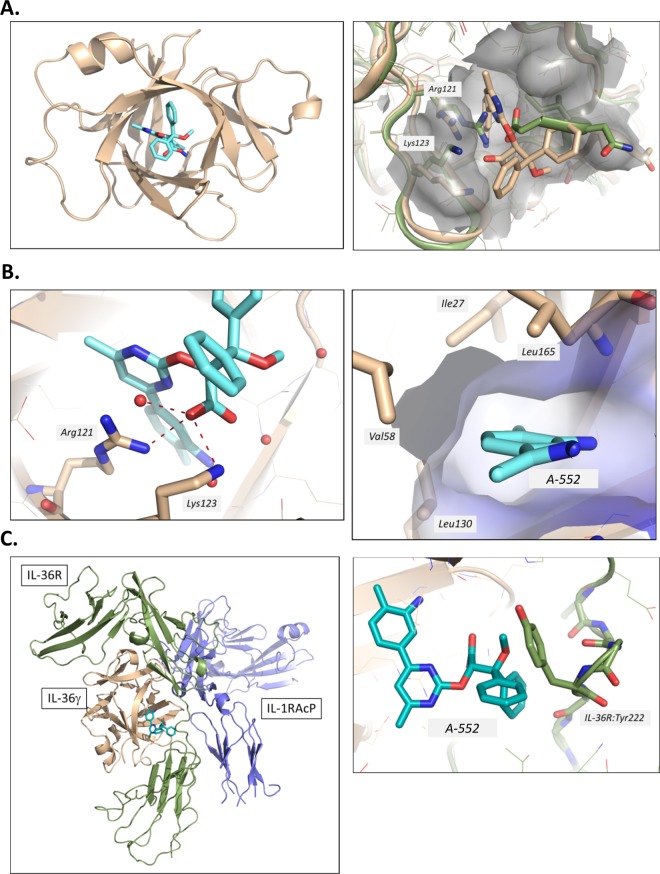
Crystal Structure of A-552 bound to human IL-36γ. (**A**) Ribbon diagram of the crystal structure of A-552 shown in cyan bound to IL-36γ shown in tan (left). Overlay of apo IL-36γ (PDB: 4IZE, shown in green) and ligand bound IL-36γ illustrates the movement of the C-terminal tail and side chain conformational changes to Arg121 and Lys123 (right). (**B**) A-552 is bound in the pocket with several stabilizing interactions to IL-36γ: Ligand carboxylate is bound to Arg121 and Lys123 (left) and hydrophobic occupation of the back pocket which is created by IL-36γ residues Ile27, Leu165, Val58 and Leu130 (right). (**C**) Model of the IL-36γ (tan)/IL-36R (green)/IL-1RAcP(purple) ternary complex with respect to A-552 (in cyan) binding. A-552 binds in a region where IL-36R interacts with IL-36γ through its D3-D1D2 linker segment (left). Close up view of linker segment and IL-36R Tyr222 which would not able to insert into the IL-36γ binding cavity upon A-552 binding (right).

The co-crystal structure of human IL-36γ and A-552 was determined to 2.0 Å resolution. When comparing the structure to the previous apo solved structure of IL-36γ^22^, the r.m.s.d is calculated to be 0.85 Å. In the new IL-36γ structure, A-552 is bound at the center of the cytokine’s β-trefoil fold (Fig. 5A) with a calculated pocket volume of 150 Å^3^. When comparing the apo and ligand bound structures, the C-terminal tail moves in order to open the pocket for ligand binding (Fig. 5A). There are several important stabilizing interactions to note. Firstly, the carboxylate at the center of the ligand makes key hydrogen bond interactions to residues Arg121 and Lys123 (Fig. 5B). Arg121 also provides a π-cation stacking interaction to the central ligand pyrimidine ring. Additionally, the methyl aniline inserts into the back of the ligand pocket in a hydrophobic space which is outlined by residues Val58, Leu130, Leu165 and Ile27 (Fig. 5B). The structure demonstrates why A-552 likely has a higher affinity than A-553 as A-552 presents the carboxylate in an optimal orientation to engage basic residues Arg121 and Lys123 with respect to the flanking phenyl rings. In this binding pose, less active enantiomer A-553 would not be able to make the necessary hydrogen bonds to these residues due to lack of proximity. This x-ray structure also rationalizes why A-552 does not show affinity for either mouse IL-36γ, human IL-36α or IL-36β. Firstly, from sequence comparison, mouse IL-36γ and human IL-36α/β have different residues at the position analogous to Arg121 in human IL-36γ (mouse IL-36γ: His116, IL-36α/β: His109/Thr108). Histidine and threonine at these positions would not be able to interact with the ligand carboxylate due to long non-optimal distances between the residue side chains and the ligand carboxylate oxygen. Secondly, the back pockets of mouse IL-36γ and human IL-36α contain Phe125 and Phe118 respectively, in contrast to Leu130 in human IL-36γ. This residue difference creates a shallower binding pocket in mouse IL-36γ and human IL-36α due to the larger side chain of phenylalanine which would prevent A-552’s methyl aniline insertion (Supplementary Fig. 5A). Finally, human IL-36β contains a glutamate residue (Glu116) in contrast to Ser128 in IL-36γ. Glu116 introduces a negative charge in the binding pocket which would clash with the negative charge of the carboxylic group from A-552 (Supplementary Fig. 5B). The terminal biphenyl group does not play any specific role in the ligand binding to IL-36γ; however, it projects into the space where IL-36R would be in the IL-36γ/IL-36R/IL-1RAcP ternary complex. A model of IL-36R bound to IL-36γ and IL-1RAcP was created using the crystal structure of the IL-1R complex as a template^45^. This model demonstrated why the IL-36γ ligand binding pocket can inhibit heterodimeric assembly (Fig. 5C). The trajectory of the terminal biphenyl group can sterically occlude the IL-36R linker (connecting the D3 and D1/D2 domains) from binding to IL-36γ ligand. Specifically, the IL-36R Tyr222 side chain would not be able to insert into the IL-36γ binding cavity (Fig. 5C).

### Human IL-36γ induced acute pro-inflammatory chemokine release in mouse plasma

**Figure 6:**
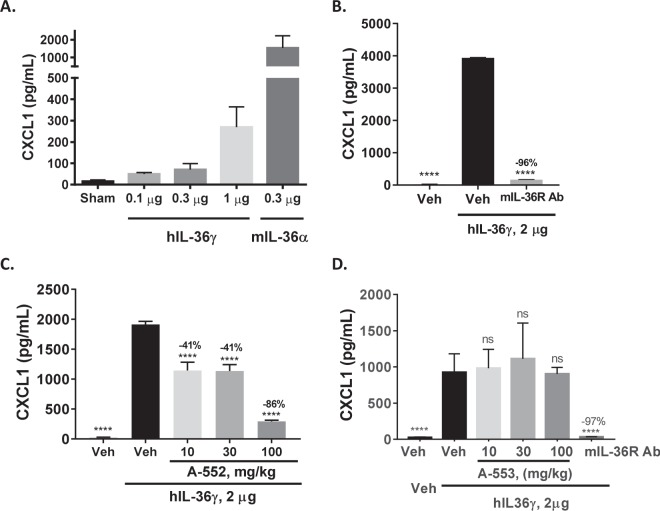
Effects of A-552 on hIL-36γ-induced CXCL1 release in mice. (**A**) hIL-36γ induced CXCL1 release in a dose dependent manner (mIL-36α was used as a positive control). (**B**) hIL-36γ-induced CXCL1 release was completely blocked by anti-mouse IL-36R antibody. (**C**) hIL-36γ-induced CXCL1 release was inhibited by A-552 in a dose-dependent manner. (**D**) A-553 had little effect on CXCL1 release induced by hIL-36γ. Average of 6 mice per group is shown ± S.E.M. (****p < 0.0001 using ANOVA with Dunnett’s post hoc test vs. Veh + hIL-36γ; (F; DF) = 46732; 17 (**B**), 78.41; 28 (**C**), 22.77; 34 (**D**)).

Previous studies have shown that subcutaneous injection of mouse IL-36α induced acute release of CXCL1 in plasma^15^. Despite the low sequence homology between human and mouse IL-36γ (60% identity, Supplementary Fig. 6), *in vitro* studies above have shown that human IL-36γ can activate the mouse IL-36R pathway inducing chemokine production from the mouse fibroblast cell line (Fig. 2D). To determine if this species cross reactivity translated to *in vivo* studies human IL-36γ was subcutaneously injected into mice. Significant levels of CXCL1 were detected in plasma 2 h post injection suggesting that human IL-36γ retained its cross-species activity *in vivo* (Fig. 6A). To define that this is occurring specifically through engagement of the IL-36 signaling pathway a mouse IL-36R antagonistic antibody^15^ was dosed 20–24 h prior to injection of the human IL-36γ. Blocking IL-36R completely attenuated CXCL1 release (Fig. 6B) supporting target engagement in this model system.

### A-552 attenuates human IL-36γ induced chemokine release in mouse plasma

The ability of enantiomers A-552 and A-553 to attenuate human IL-36γ induced plasma CXCL1 was assessed in an acute mouse model. Significant CXCL1 plasma levels were induced in the vehicle control arm following subcutaneous administration of human IL-36γ (Fig. 6C). Pretreatment with A-552 robustly attenuated CXCL1 levels in plasma in a dose-dependent manner. In contrast, the less active enantiomer had no effect on the CXCL1 levels induced by human IL-36γ up to 100 mg/Kg demonstrating the higher potency of A-552 with respect to interaction with hIL-36γ in an *in vivo* setting (Fig. 6D).

### A-552 attenuates the human IL-36γ induced 3D skin equivalent model of inflammation

**Figure 7:**
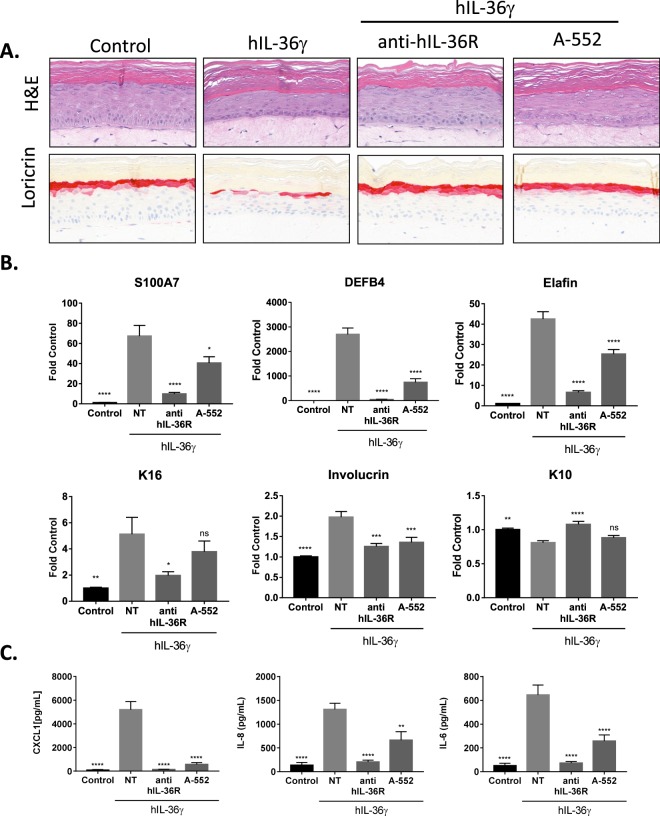
A-552 attenuates hIL-36γ induced inflammation in human 3D skin equivalents. (**A**) Staining of fully differentiated 3D skin equivalents untreated or treated with 0.3 μg/mL hIL-36γ alone or in the presence of function blocking 10 μg/mL anti-hIL-36R or 10 μM A-552. Representative images at 32X magnification are shown. (**B**) Differences between RNA transcript levels in treated and non-treated 3D skin equivalents were quantified for each probe using qRT-PCR. Average of 6 treated and 7 control samples is shown ± S.E.M. (*p < 0.05, **p < 0.01, ***p < 0.001, ****p < 0.0001 using ANOVA with Dunnett’s post hoc test vs. hIL-36γ alone; (F; DF) = 26.05; 24 (S100A7), 75.76; 24 (DEFB4), 82.13; 24 (Elafin), 6.221; 24 (K16), 19.53; 23 (Involucrin), 13.16; 24 (K10)). (**C**) Differences between secreted CXCL1, IL-8 and IL-6 proteins in treated and non-treated 3D skin equivalents were quantified using immunoassays. Average of 6 treated (5 in CXCL1 and IL-8 NT column) and 7 control samples is shown ± S.E.M. (**p < 0.01, ****p < 0.0001 using ANOVA with Dunnett’s post hoc test vs. hIL-36γ alone; (F; DF) = 61.15; 23 (CXCL1), 21.31; 23 (IL-8), 32.46; 24 (IL-6)).

As demonstrated above (Fig. 1C–E) human IL-36γ induces morphological as well as changes in gene and protein expression in 3D human skin equivalents that correlate well with changes observed in PV. To demonstrate the effectiveness of A-552 on psoriasis-like changes evoked by human IL-36γ treatment, human 3D skin equivalents were treated with human IL-36γ alone and in conjunction with A-552 or anti-human IL-36R antibody as a positive control. Human IL-36γ induced changes in morphology (such as hypogranulosis and loss of the terminal differentiation marker Loricrin), gene expression and cytokine production were attenuated by treatment with either A-552 or anti-human IL-36R (Fig. 7A–C; Supplementary Fig. 7). A-552 and other human IL-36γ antagonists had no appreciable effect on 3D human skin equivalents when treated alone, suggesting that their action is not the result of off-target effects (Supplementary Fig. 8).

## Discussion

IL-36γ is consistently elevated in psoriatic lesions and potentially contributes to the pathophysiology of PV (Fig. 1)^8,13^. Attenuating IL-36 signaling with a small molecule inhibitor could provide moderate-to-severe psoriasis patients with an alternate therapy to currently clinically approved therapies such as the oral PDE4 inhibitor apremilast or the systemic biologics treatments. Following apremilast the next generation oral small molecule therapy approaches for psoriasis patients are in early clinical development and are focused on directly modulating the IL-23/Th17 axis^3,46,47^. While these approaches hold much promise for robust efficacy like that observed with biologic therapies one of the major challenges remaining will be achieving sufficient safety margins to support chronic treatment. It has been recently shown in a study of individuals carrying loss of function mutations in the IL-36R gene that attenuated IL-36 signaling does not lead to compromised host defense and normal immune function is retained^48^ suggesting that targeting the IL-36R signaling pathway may provide a therapy with a good safety profile.

Directly targeting inhibition of the IL-36 signaling pathway through cytokine/cytokine receptor disruption was a challenging approach. An alternative strategy to inhibit this pathway would be to target one of the enzymes that cleave and activate the IL-36 cytokines when they are expressed in the skin^40,49,50^. IL-36α, IL-36β and IL-36γ are activated following proteolytic cleavage at the N-terminus by the neutrophil granule-derived proteases cathepsin G, elastase and proteinase-3 or by keratinocyte/fibroblast derived Cathepsin S. Recently, small molecule inhibitors of elastase that can prevent activation of IL-36γ were identified by *in silico* screening^49^. These inhibitors (exemplified by LCB016) can attenuate elastase driven processing of full length IL-36γ and the downstream pro-inflammatory signaling induced by fully active IL-36γ^49^. However, safety concerns regarding systemic use of such inhibitors in psoriatic patients remains to be addressed as elastase is important for normal neutrophil mediated antibacterial responses^51,52^.

This study reports the identification of a series of first in class small molecule human IL-36γ inhibitors that functionally attenuate the activation of the IL-36R signaling pathway in cellular and *in vivo* models of inflammation. To achieve this several different assay platforms were interrogated. Initially cell based assays were considered including a novel GlycoFRET binding assay platform^44^ and a cell-based hIL-36γ induced IL-8 release assay. Unfortunately, despite both assays having excellent dynamic range these formats were not optimal for screening the compound library at high drug concentrations due to the limitations in DMSO tolerability in a cellular setting. Additionally, hits identified from the functional cell-based assay would need significant hit triage as a positive hit in the screen could be a result of modulating any of the proteins in the downstream signaling cascade. To circumvent these issues a novel TR-FRET biochemical assay was developed as the primary screen to identify molecules that could block human IL-36γ binding to the human IL-36R through binding either the receptor or the cytokine directly. Based on earlier studies it was known that to drive high affinity binding of IL-36 to its cognate receptor required the presence of both IL-36R and IL-1RAcP proteins^28^, therefore a key requirement for the assay was the expression IL-36R/IL-1RAcP ECD heterodimer^22^. This was achieved by engineering both proteins with an Fc “knob-in-hole” to force heterodimerization. This novel TR-FRET binding assay led to the identification of the hit A-706 that binds human IL-36γ and functionally antagonizes the IL-36R driven signaling. Four other hits from three different series were also identified (not shown) and could serve as a starting point for future medicinal chemistry efforts.

One of the challenges for targeting the IL-36R signaling pathway directly arises as a result of the low amino acid sequence identity between human and mouse IL-36γ (60%) and IL-36R (66%)^53,54^ (Supplementary Fig. 6). A-552 binds with high affinity to human IL-36γ but not mouse IL-36γ. Data herein showed that human IL-36γ can activate both human IL-36R and mouse IL-36R similarly thereby enabling the assessment of this novel antagonist in mouse *in vivo* studies driven directly by exogenously delivered human IL-36γ. Exploiting this species cross-reactivity has led to the development of a novel human IL-36γ induced CXCL1 release assay in mouse plasma. The more potent enantiomer A-552 attenuated human IL-36γ induced CXCL1 release in a dose-dependent fashion while the less active enantiomer (A-553) did not have any effects up to 100 mg/Kg dosed orally despite both enantiomers achieving similar drug plasma levels.

Unfortunately, the lack of species cross-reactivity of A-552 to mouse IL-36α or IL-36γ precluded assessing the effects of this inhibitor in preclinical mouse models of psoriasiform dermatitis induced by injection of IL-23 or the TLR7 agonist IMQ. The IL-23 model has been reported to have the closest skin transcriptomics profile to human PV^55,56^ including elevated levels of IL-36 cytokines that can be attenuated by mouse IL-36R antibodies^15^. It has also been shown that inhibition of IL-36R signaling with antagonistic antibodies attenuated ear thickness and psoriasis target gene signature in the IMQ model of skin inflammation^48,57^.

To provide enhanced validation of the role of IL-36γ in skin inflammation an *in vitro* human model of psoriasis was established by treating 3D skin equivalents with hIL-36γ similar to a previously reported model^40,41^. This model recapitulated many features of psoriasis including increased expression of S100A7/psoriasin, increased proinflammatory cytokine release and reduced loricrin expression that coincided with hypogranulosis as depicted by H&E staining. Pretreatment with A-552 attenuates all these disease features, albeit not to the same extent as IL-36Ra and anti-IL-36R antibody, with no apparent off-target effects suggesting that 2-oxypyrimidine series is a viable candidate for further medicinal chemistry efforts to enhance potency.

Collectively, these studies have led to the discovery of A-552, a novel first in class small molecule antagonist of the IL-36 signaling pathway that binds selective human IL-36γ and can dose-dependently inhibit IL-36γ induced production of proinflammatory cytokines from human and mouse cells. This inhibitor also attenuated an IL-36γ induced psoriasis phenotype in a 3D human skin equivalent model. This orally bioavailable IL-36γ antagonist has the potential to serve as a robust tool to study IL-36 biology and IL-36R signaling in the laboratory models of inflammatory diseases including psoriasis.

## Supplementary information

Supplementary Info
