# Supplementary material for: Small Molecule IL-36γ Antagonist as a Novel Therapeutic Approach for Plaque Psoriasis
Source: Sci Rep. 2019 Jun 24;9:9089. doi: 10.1038/s41598-019-45626-w (PMC6591177; doi:10.1038/s41598-019-45626-w)

Small Molecule IL-36γ Antagonist as a Novel Therapeutic Approach for Plaque Psoriasis

Viktor Todorović[1], Zhi Su[1], C. Brent Putman[1], Stevan J. Kakavas[1], Katherine M. Salte[1], Heath A. McDonald[1], Joseph B. Wetter[1], Stephanie E. Paulsboe[1], Qi Sun[1], Clare E. Gerstein[1], Limary Medina[2], Bernhard Sielaff[2], Ramkrishna Sadhukhan[2], Henning Stockmann[1], Paul L. Richardson[1], Wei Qiu[1], Maria A. Argiriadi[2], Rodger F. Henry[1], J. Martin Herold[2], J. Brad Shotwell[1], Steve P McGaraughty[1], Prisca Honore[1], Sujatha M. Gopalakrishnan[1], Chaohong C. Sun[1] and Victoria E. Scott[1]

[1]AbbVie Inc., 1 North Waukegan Rd., North Chicago, IL 60064; [2]AbbVie Bioresearch Center, 381 Plantation St., Worcester, MA 01605

Supplementary Figure 1: In situ hybridization controls. The positive (PPIB, right) and negative (DapB, left) control probe slides showed expected staining patterns in lesional skin.

Supplementary Figure 2: Quantification of treatments on 3D skin equivalents with hIL-36γ. Quantification of raft histology was captured by analyzing the area of positive loricrin staining normalized to the length of the raft section. Average of 6 samples is shown ± S.E.M. (*p<0.05, ****p<0.0001 using ANOVA with Dunnett's post hoc test vs. hIL-36γ alone, (F; DF)= 13.57; 29).

Supplementary Figure 3: Validation of IL-36γ in functional assays. A. Quantification of unlabeled (left, n=3) and Alexa-488 labeled (right, n=3) hIL-36γ activity in evoking IL-8 release from HaCaT keratinocytes. **B.** Mouse-to-human cross-species activity of mIL-36γ was measured by its inability to induce the secretion of CXCL1 from human HaCaT keratinocytes (n=2). All data represents an average ± S. E. M.

Supplementary Figure 4: Active compound series do not bind to hIL-36α. Thermal shift assay for hIL-36α in the absence (black) and in the presence of 100 μM of A-706 (green), A-552 (purple) and A-553 (blue).

Supplementary Figure 5: Structure comparison of A-552 binding pockets among IL-36 family members. A. An overlay of human IL-36γ x-ray structure (tan) with homology models of human IL-36α (blue) and mouse IL-36γ (grey) shows key residue differences (Leu → Phe, Arg → His) in the binding pocket and rationalizes why A-552 does not have

affinity for mouse IL-36γ and human IL-36α. **B.** An overlay of human IL-36γ x-ray structure (tan) with homology model of human IL-36β (magenta) shows key residue differences (Ser → Glu, Arg → Thr) in the binding pocket and rationalizes why A-552 does not have affinity for human IL-36β.

Supplementary Figure 6: Amino acid sequence alignment of select IL-36 family members. (Top) IL-36γ alignment, (Bottom) IL-36R alignment. Green – identical amino acids; Orange – similar amino acids.

Supplementary Figure 7: Isotype control treatment has no effect on hIL-36γ induced psoriasis-like phenotype in 3D skin equivalents. A. Staining of fully differentiated 3D skin equivalents untreated or treated with 0.3 μg/mL hIL-36γ with or without 10 μg/mL Isotype control. Representative images at 32X magnification are shown. **B.** Differences between RNA transcripts in treated and non-treated 3D skin equivalents were quantified for each probe using qPCR. Average of 7 control, 6 hIL-36γ (except for K16 where 3 samples were available), 3 hIL-36γ + isotype control and 4 isotype control samples is shown ± S.E.M. (**$p<0.01$, ***$p<0.001$, ****$p<0.0001$ using ANOVA with Dunnett's post hoc test vs. hIL-36γ alone; (F; DF)= 32.56; 19 (S100A7), 108.2; 19 (DEFB4), 136.9; 19 (Elafin), 37.97; 16 (K16), 28.8; 19 (Involucrin), 7.639; 19 (K10)). **C.** Supernatants were harvested 48 hours after treatment and CXCL1, IL-6, and IL-8 were quantified**.** Average of 7 controls, 5 hIL-36γ, 2 hIL-36γ + isotype control and 4 isotype control samples is shown ± S.E.M. (****$p<0.0001$ using ANOVA with Dunnett's post hoc test vs. hIL-36γ alone; (F; DF)= 62.75; 17 (CXCL1), 24.1; 17 (IL-8), 32.59; 17 (IL-6)).

Supplementary Figure 8: IL-36 antagonists have little effect on 3D skin equivalents on their own. A. Staining of fully differentiated 3D skin equivalents untreated or treated with function blocking 10 μg/mL anti-hIL-36R, 10 μg/mL hIL-36Ra, or 10 μM A-552. Representative images at 32X magnification are shown. **B.** Differences between RNA transcripts in treated and non-treated 3D skin equivalents were quantified for each probe using qPCR. Average of 8 control and 5 treatment samples is shown ± S.E.M. (*$p<0.05$ using ANOVA with Dunnett's post hoc test vs. control; (F; DF)= 0.81; 22 (S100A7), 2.31; 22 (DEFB4), 1.67; 22 (Elafin), 1.53; 22 (K16), 4.95; 22 (Involucrin), 0.86; 22 (K10)). **C.** Supernatants were harvested 48 hours after treatment and CXCL1, IL-6, and IL-8 were quantified**.** Average of 7 control and 5 treatment samples is shown ± S.E.M. (no significant differences found using ANOVA with Dunnett's post hoc test vs. control; (F, DF)= 1.01; 21 (CXCL1), 1.28; 21 (IL-8), 0.82; 21 (IL-6)).

Supplementary Table 1: Summary High Throughput Screening.

Supplementary Table 2: Crystallographic statistics for the x-ray structure of IL-36γ : A-552 complex.

Supplementary Table 1

Category	Parameter	Description
Assay	Type of assay	*In vitro*, TR-FRET
	Target	huIL-36R Signaling
	Primary measurement	Fluorescence Ratio (520 nm/ 495 nm)
	Key reagents, final concentrations	a. Assay buffer = DPBS, Gibco # 14190-144 + 0.1% BSA, Gibco # 15260-037.<br>b. 0.3 ug/ml IL-36 Receptor = knob-in-a-hole huIL-36R/huIL-1RAcP. (in-house)<br>c. 2 nM Tb-anti-His Antibody, Life tech. Part# PV5895.<br>d. 4 nM hIL-36g-Alexa488. (in-house)
	Assay protocol	1. Prepare **2X receptor** by diluting stock to 0.6 ug/ml in assay buffer. Use Multi-Drop Combi to add **2.5 ul** to each well of ProxiPlate containing 30 nL compound in DMSO.<br>2. Incubate assay plate at RT for **60 minutes**.<br>3. Prepare **2X detection mixture** by diluting Tb-anti-His stock to 4 nM and hIL-36g-Alexa488 stock to 8 nM together in assay buffer.<br>4. Use Multi-Drop Combi to add **2.5 ul** of 2X detection mix to each well of assay plate (total volume = 5.0 ul).<br>5. Incubate assay plate at RT in the dark for **4 hours**.<br>6. Read on Envision (excitation 320, emission 520:495).
	Additional comments	N/A
Compound Library	Library size	850,000
	Library composition	Collection of commercially available & proprietary compounds
	Source	Commercial and internal
Screen	Format	384 well PE Proxiplates, 1 compound per well
	Concentration(s) tested	30 uM
	Plate controls	No receptor = positive control for 100% inhibition<br>DMSO only = negative control for 0% inhibition
	Reagent/ compound dispensing system	Echo Liquid Handler for compound dispense.<br>Multi-drop Combi for all other reagents.
	Detection instrument and software	Perkin Elmer Envision<br>HTS Database
	Assay validation/QC	Z', S/N
	Correction factors	None
	Normalization	Data reported as % inhibition
	Additional comments	N/A

Post-HTS analysis	Hit criteria	>50% inhibition at 30 uM & concentration response curve for confirmation
	Hit rate	542 out of 850,000 compounds = 0.06%
	Additional assay(s)	Further confirmation assays described in detail in this manuscript.
	Confirmation of hit purity and structure	Compounds were verified for identity by mass spectrometry and NMR spectroscopy. Purity was established by HPLC with UV detection at 220 and 254 nM. Compounds were later resynthesized for confirmation.
	Additional comments	N/A

Supplementary Table 2

Structure	IL-36γ complexed to A-552
**PDB code**	6P9E
**Data Collection**	
Resolution (Å)	39.4–2.00
Space Group	I23
Unit Cell Lengths (a=b=c; Å)	96.4
Unique reflections	9984
**Overall Statistics**	
R_{sym} (%)	0.065 (0.57)

I/σ_I	28.8 (6.2)
Data completeness (%)	97.7 (100.0)
Mean multiplicity	18.4(19.0)
**Refinement**	
Reflections used in refinement	9982
R_{cryst} (%)	21.7
R_{free} (%)	24.2
R.m.s. deviations, bond lengths (Å), bond angles (°)	0.010, 1.17

Supporting Information, Chemistry

General considerations:

The starting materials 2-hydroxy-3-methoxy-3,3-diphenylpropanoic acid and 4-chloro-6-methyl-2-(methylthio)pyrimidine are commercially available from Shanghai Bide Pharmaceuticals Corp. and used without further purification. All other commercially obtained reagents were used as received.

^{1}H-NMR spectra were recorded on a Bruker 400 MHz Advance III. LCMS was performed on Shimadzu LC-20AD+MS2020 and Agilent 1200 series 1956A MS.

Synthesis Route:

Scheme SI-1

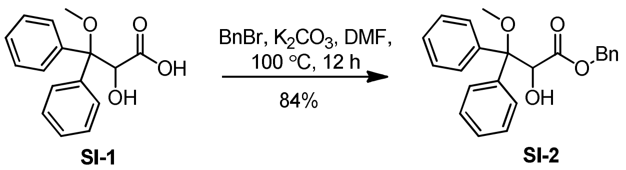

Scheme SI-2

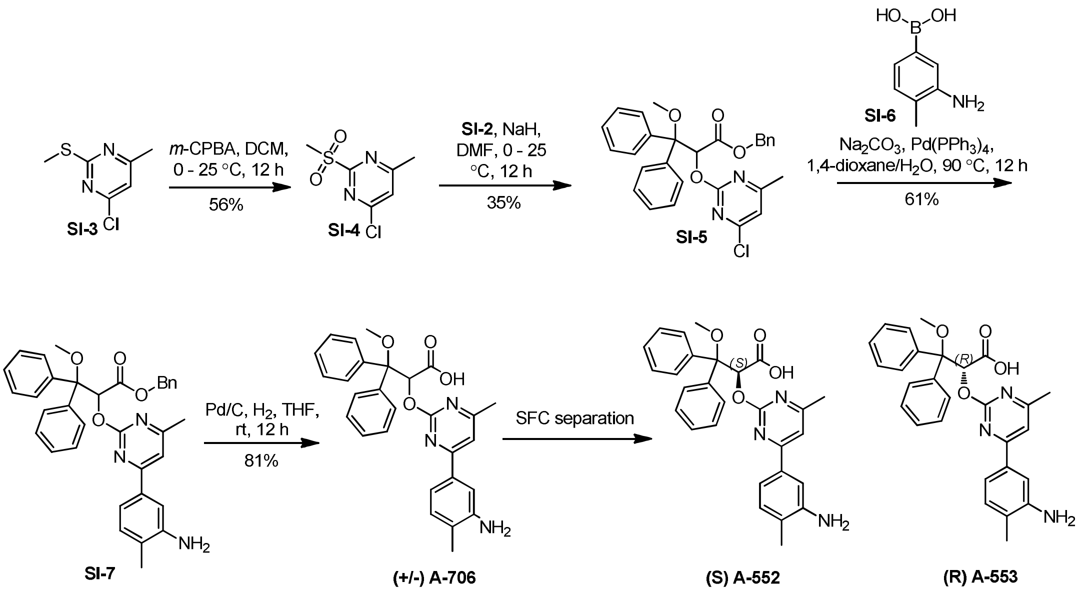

Detailed synthetic procedures:

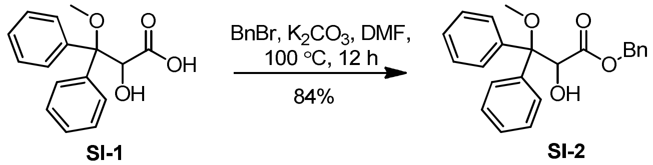

Preparation of benzyl 2-hydroxy-3-methoxy-3,3-diphenylpropanoate (SI-2):

To a solution of 2-hydroxy-3-methoxy-3,3-diphenylpropanoic acid (40 g, 147 mmol) in DMF (400 mL) was added potassium carbonate (22.3 g, 162 mmol) at 25 °C. The reaction mixture was stirred at 25 °C for 1 h. Benzyl bromide (17.5 mL, 147 mmol) was added to the reaction mixture at 25 °C. The reaction mixture was stirred at 100 °C for 12 h. The reaction mixture was treated with water (800 mL) and extracted with dichloromethane (3 × 400 mL). The combined organic layers were washed with brine (500 mL), dried over Na_2SO_4, filtered, and concentrated to give a residue which was purified by flash chromatography on silica gel (petroleum ether: ethyl acetate = 100 : 1 to 5 : 1) to give the title compound **SI-2** (44 g, yield 84%) as white solid.

^{1}H NMR (400 MHz, Methanol-d_4) δ 7.39 – 7.15 (m, 15H), 5.30 (s, 1H), 4.99 (s, 2H), 3.16 (s, 3H).

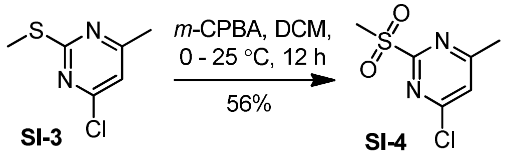

Preparation of 4-chloro-6-methyl-2-(methylsulfonyl)pyrimidine (SI-4):

To a solution of 4-chloro-6-methyl-2-(methylthio)pyrimidine **SI-3** (20 g, 115 mmol) in DCM (500 mL) was added *m*-CPBA (93 g, 458 mmol) at 0 °C. The reaction mixture was stirred at 25 °C for 12 h. The reaction mixture was added into an aqueous solution of NaHCO3 (350 mL) dropwise. The suspension was filtered and the filter cake was washed with DCM (200 mL). The combined organic layer was washed with brine (150 mL), dried over Na_2SO_4, filtered and concentrated to afford **SI- 4** (14 g, yield 56%) as white solid.

^{1}H NMR (400 MHz, Chloroform-*d*) δ 7.42 (d, *J* = 0.7 Hz, 1H), 3.36 (s, 3H), 2.67 (d, *J* = 0.6 Hz, 3H).

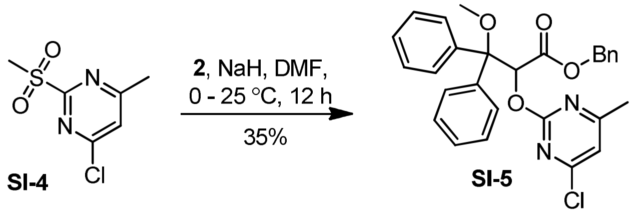

Preparation of benzyl 2-((4-chloro-6-methylpyrimidin-2-yl)oxy)-3-methoxy-3,3-diphenylpropanoate (SI-5):

To a solution of benzyl 2-hydroxy-3-methoxy-3,3-diphenylpropanoate **SI-2** (21.9 g, 60.5 mmol) in DMF (300 mL) was added NaH (3.63 g, 91 mmol) at 0 °C. The reaction mixture was stirred at 0 °C for 1 h and then 4-chloro-6-methyl-2-(methylsulfonyl)pyrimidine SI-4 (12.5 g, 60.5 mmol) was added to the reaction mixture and the reaction was stirred at 25 °C for 12 h. The reaction mixture was poured into saturated aqueous NH_4Cl solution (600 mL) at 0 °C and extracted with DCM (3 × 200 mL). The organic layers were dried over Na_2SO_4, filtered and concentrated to give a residue which was purified by flash chromatography on silica gel (petroleum ether : ethyl acetate = 20 : 1 to 5 : 1) to give **SI-5** (11 g, yield 35%) as white solid.

^{1}H NMR (400 MHz, Methanol-d_4) δ 7.42 – 7.37 (m, 2H), 7.37 – 7.32 (m, 2H), 7.31 – 7.16 (m, 9H), 7.08 (d, J = 0.6 Hz, 1H), 7.06 – 7.00 (m, 2H), 6.23 (s, 1H), 4.88 (d, J = 2.6 Hz, 2H), 3.39 (s, 3H), 2.32 (s, 3H).

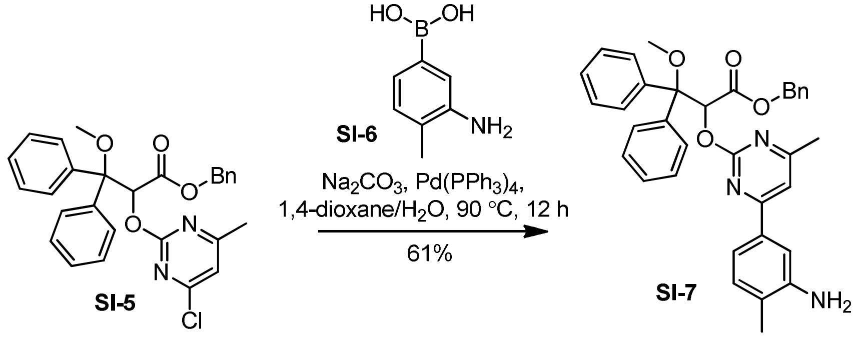

Preparation of benzyl 2-((4-(3-amino-4-methylphenyl)-6-methylpyrimidin-2-yl)oxy)-3-methoxy-3,3-diphenylpropanoate (SI-7):

To a solution of benzyl 2-((4-chloro-6-methylpyrimidin-2-yl)oxy)-3-methoxy-3,3-diphenylpropanoate **SI-5** (13.5 g, 27.6 mmol) in water (40 mL) and 1, 4-dioxane (160 mL) was added sodium carbonate (9.36 g, 88 mmol), (3-amino-4-methylphenyl)boronic acid (5.42 g, 35.9 mmol) and tetrakis(triphenylphosphine)palladium(0) (3.19 g, 2.76 mmol) at 25 °C. The reaction mixture was stirred at 90 °C for 12 h. The reaction mixture was treated with water (300 mL) and extracted with ethyl acetate (3 × 250 mL). The combined organic layer were washed with saturated $NaHCO_3$ (250 mL) and brine (150 mL), dried over Na_2SO_4, filtered, and concentrated to give a residue which was purified by flash chromatography on silica gel (petroleum ether : ethyl acetate = 100 : 1 to 5 : 1) to give **SI-7** (10 g, yield 61%) as white solid.

^{1}H NMR (400 MHz, DMSO-d_6) δ 7.45 (s, 1H), 7.41 – 7.18 (m, 15H), 7.02 – 6.93 (m, 3H), 4.98 – 4.83 (m, 4H), 3.43 (s, 3H), 2.38 (s, 3H), 2.10 (s, 3H).

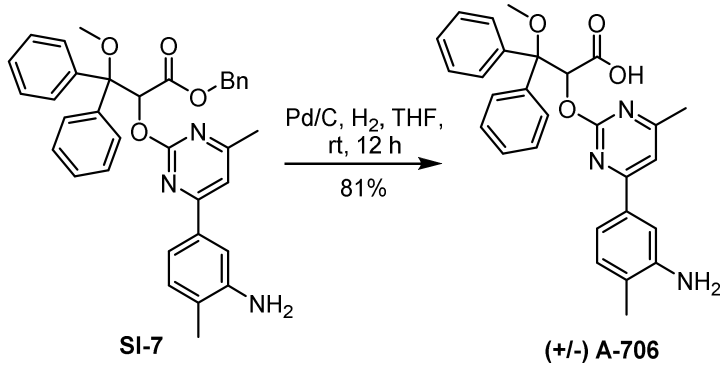

Preparation of *rac*-2-((4-(3-amino-4-methylphenyl)-6-methylpyrimidin-2-yl)oxy)-3-methoxy-3,3-diphenylpropanoic acid ((±) A-706):

To a solution of benzyl 2-((4-(3-amino-4-methylphenyl)-6-methylpyrimidin-2-yl)oxy)-3-methoxy-3,3-diphenylpropanoate 7 (10 g, 17.8 mmol) in THF (200 mL) was added Pd/C (1.9 g, 0.893 mmol) at 25 °C. The reaction was stirred at 25 °C under H_2 (15 psi) for 12 h. The reaction mixture was filtered and the filtrate was concentrated to give **(±) 1** (7 g, yield 81%) as white solid.

^{1}H NMR (400 MHz, DMSO-d_6) δ 12.54 (s, 1H), 7.45 (s, 1H), 7.42 – 7.19 (m, 12H), 7.05 (d, *J* = 7.8 Hz, 1H), 6.18 (s, 1H), 4.99 (s, 2H), 3.41 (s, 3H), 2.43 (s, 3H), 2.11 (s, 3H).

Chiral Separation:

(±) A-706 (6.9 g, 14.7 mmol) was purified by SFC to give **(S)-552** (2.65 g, yield 37%) and **(R)-553** (2.53 g, yield 36 %) eluting at 0.57 min. and 1.35 min. as yellow oils with 96.5% and 97.4% ee respectively.

Instrument: Thar SFC350 preparative SFC;

Column: Chiralpak AD-H 250 × 50mm i.d. 10 µ

Mobile phase: A for CO_2 and B for isopropanol (0.1%NH_3H_2O);

Gradient: B%=50%;

Flow rate: 200g/min;

Wavelength: 220 nm;

Column temperature: 40 °C;

System back pressure: 100 bar

(S) A-552: ^{1}H NMR (400 MHz, DMSO-d_6) δ 7.44 (s, 1H), 7.42 – 7.19 (m, 12H), 7.05 (d, *J* = 7.8 Hz, 1H), 6.18 (s, 1H), 4.98 (s, 2H), 3.41 (s, 3H), 2.43 (s, 3H), 2.11 (s, 3H). $[\alpha]_D^{20}$ +112.15 (c = 0.507, MeOH)

(R) A-553: ^{1}H NMR (400 MHz, DMSO-d_6) δ 12.55 (s, 1H), 7.44 (s, 1H), 7.41 – 7.19 (m, 12H), 7.05 (d, *J* = 7.8 Hz, 1H), 6.18 (s, 1H), 4.99 (s, 2H), 3.41 (s, 3H), 2.43 (s, 3H), 2.11 (s, 3H). $[\alpha]_D^{20}$ -110.77 (c = 0.536, MeOH)

SFC Chromatograms for both enantiomers:

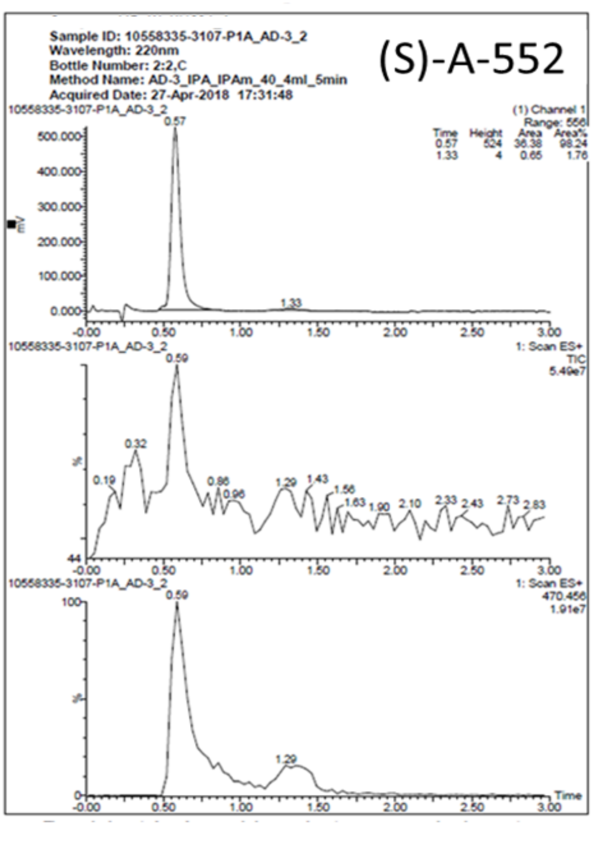

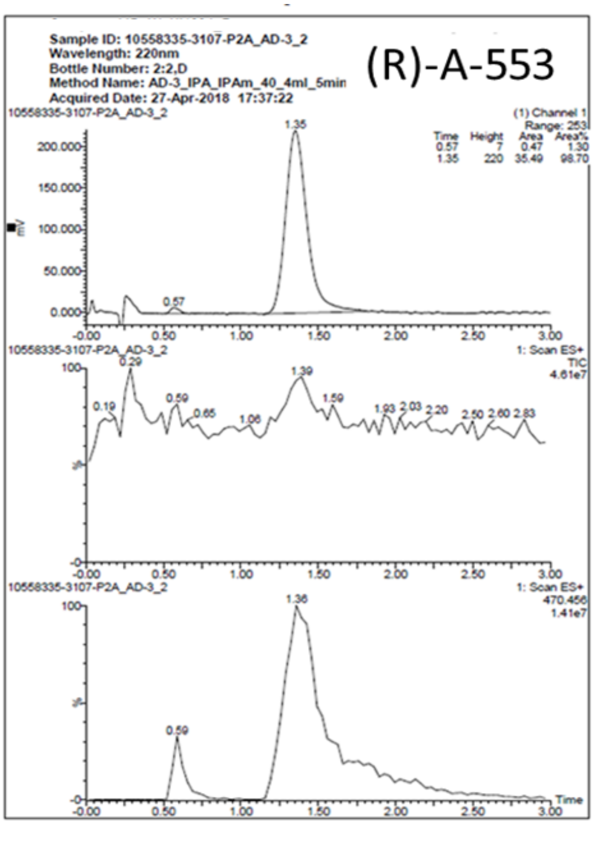

***S*-2-((4-(3-amino-4-methylphenyl)-6-methylpyrimidin-2-yl)oxy)-3-methoxy-3,3-diphenylpropanoic acid**

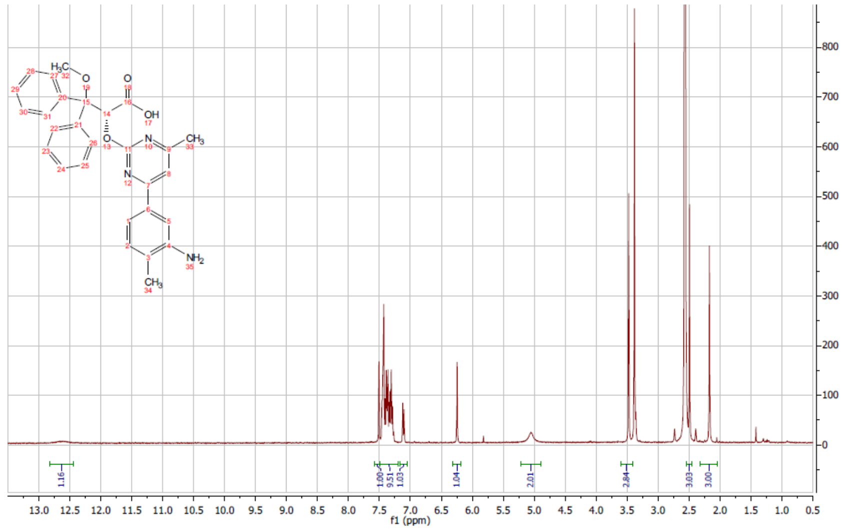

***R*-2-((4-(3-amino-4-methylphenyl)-6-methylpyrimidin-2-yl)oxy)-3-methoxy-3,3-diphenylpropanoic acid**

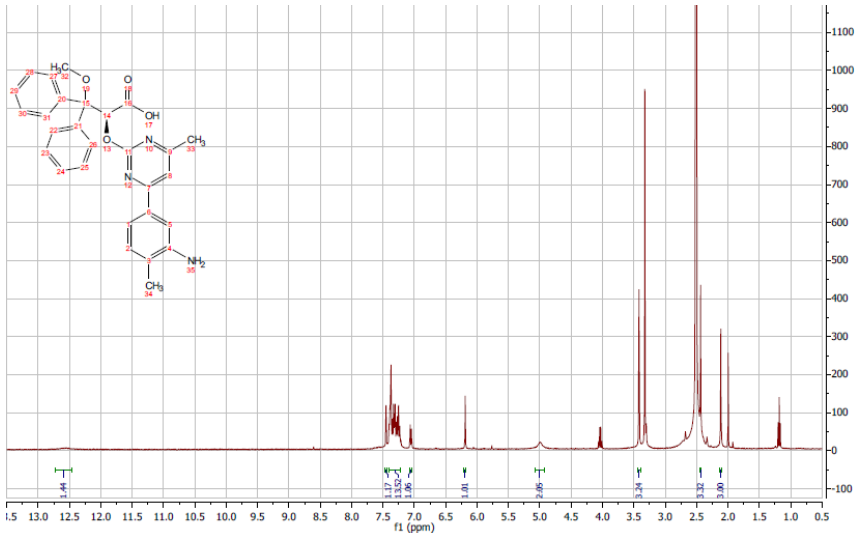

Absolute Configurations for (S)-552 and (R)-553 were unambiguously assigned by small molecule X-ray crystallography.

ORTEP (S)-552

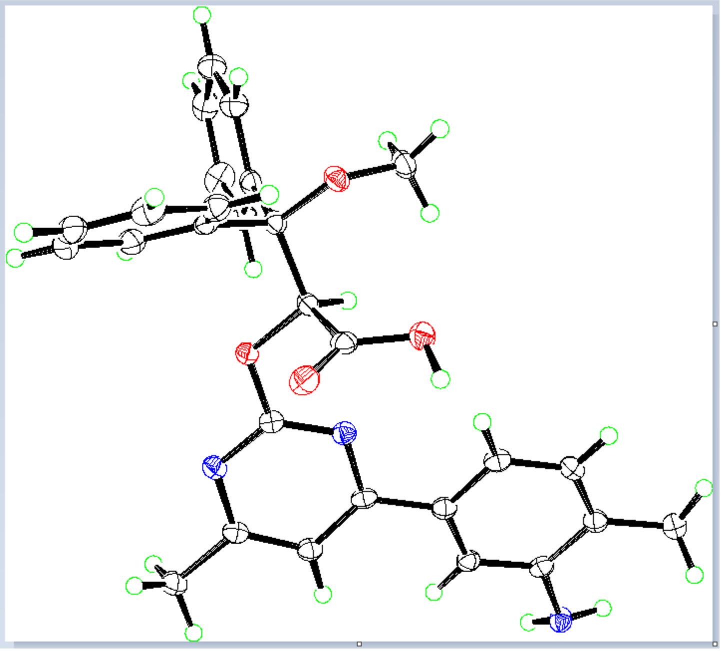

ORTEP (R)-553

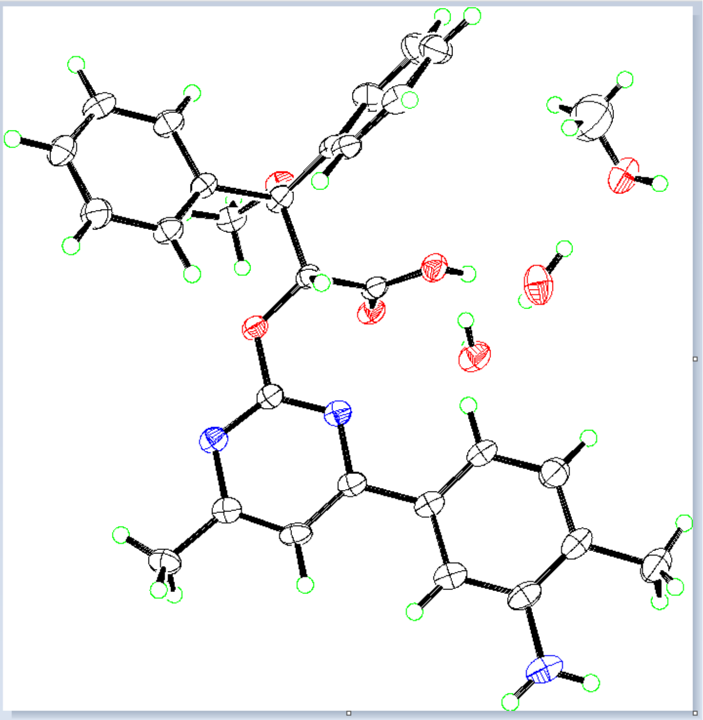

Supplementary Figure 1

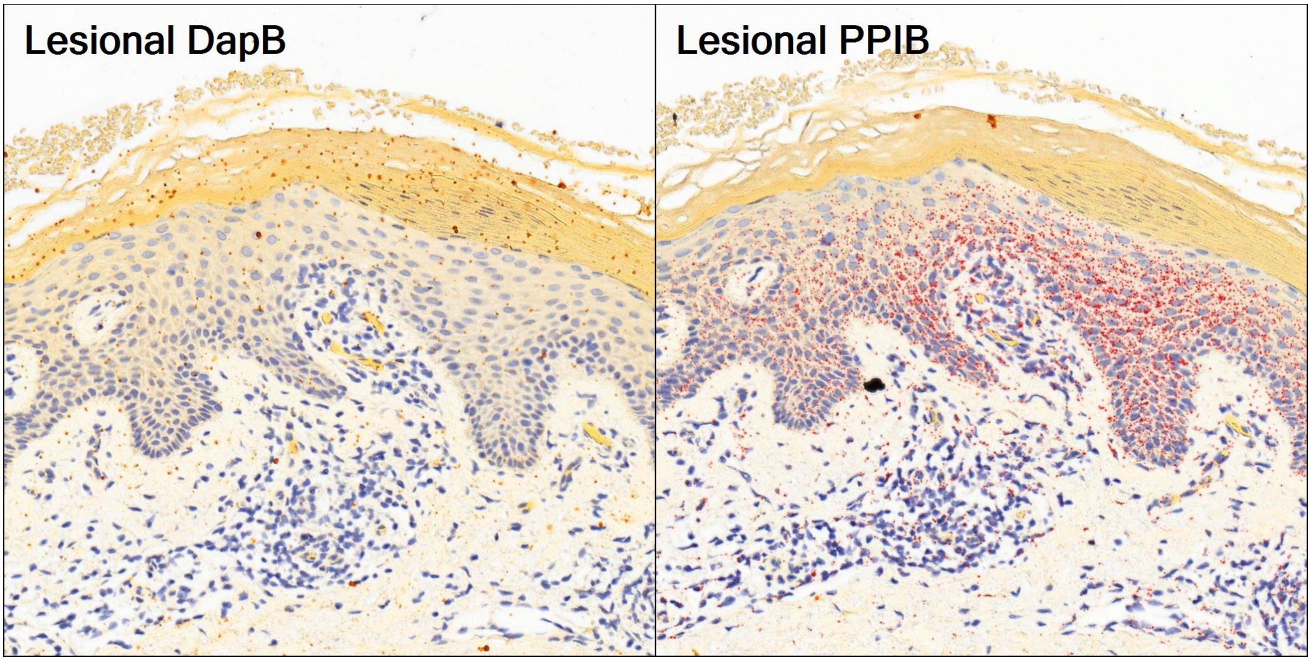

Supplementary Figure 2

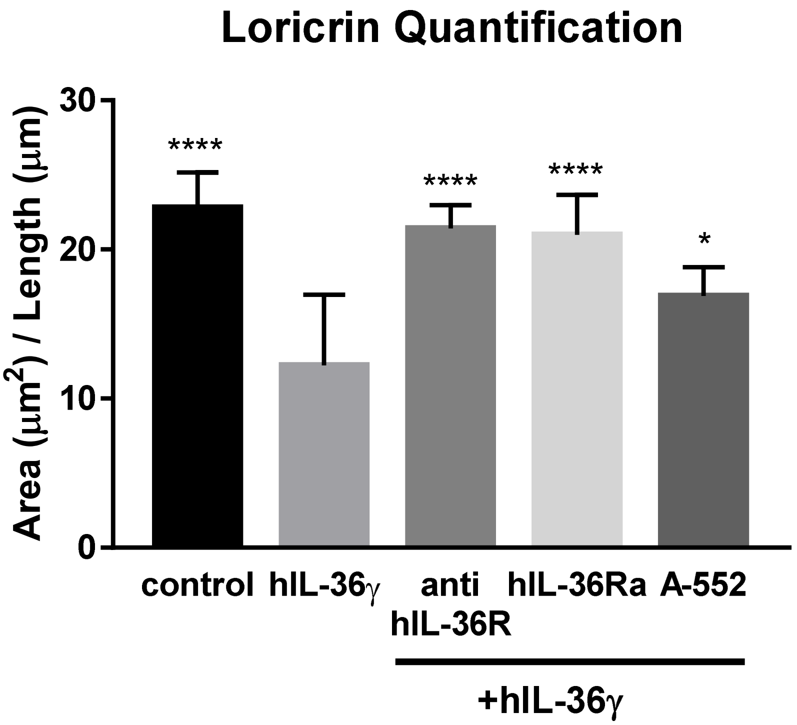

Supplementary Figure 3

A.

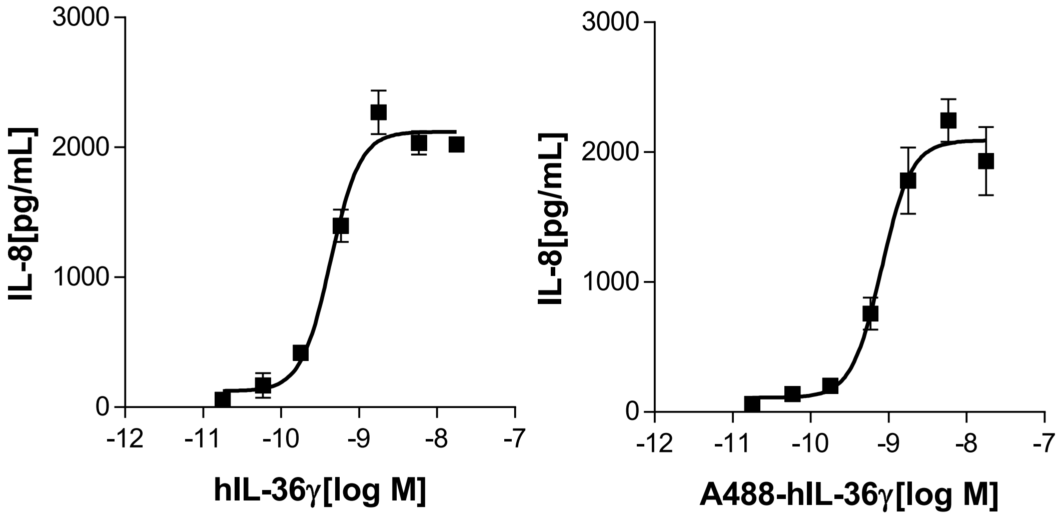

B.

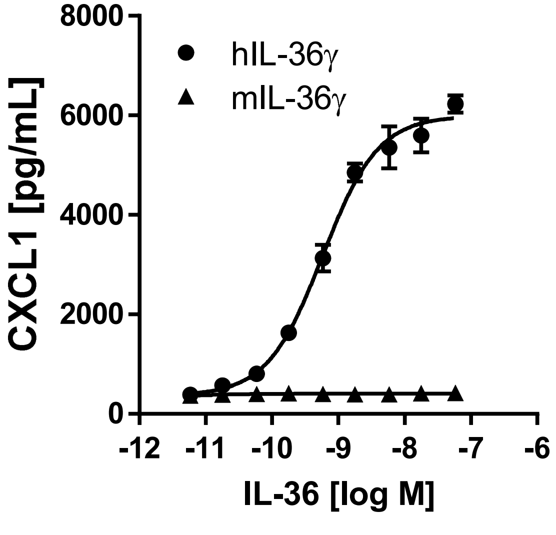

Supplementary Figure 4

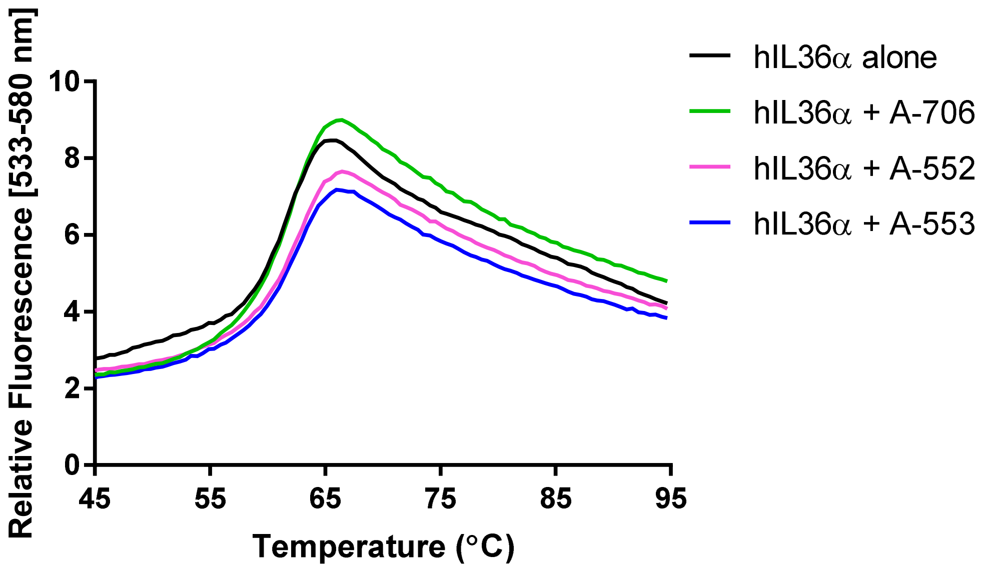

Supplementary Figure 5

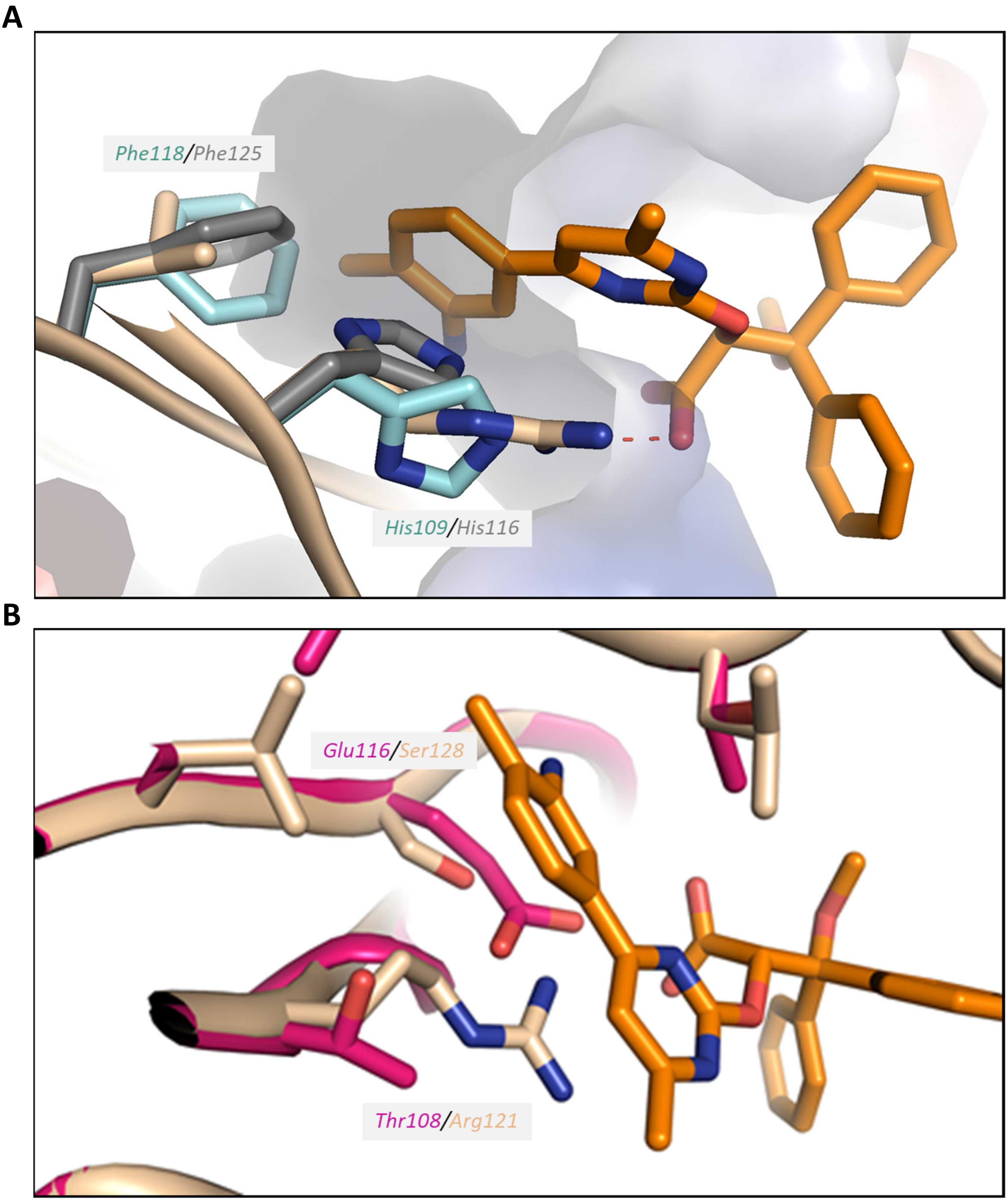

Supplementary Figure 6

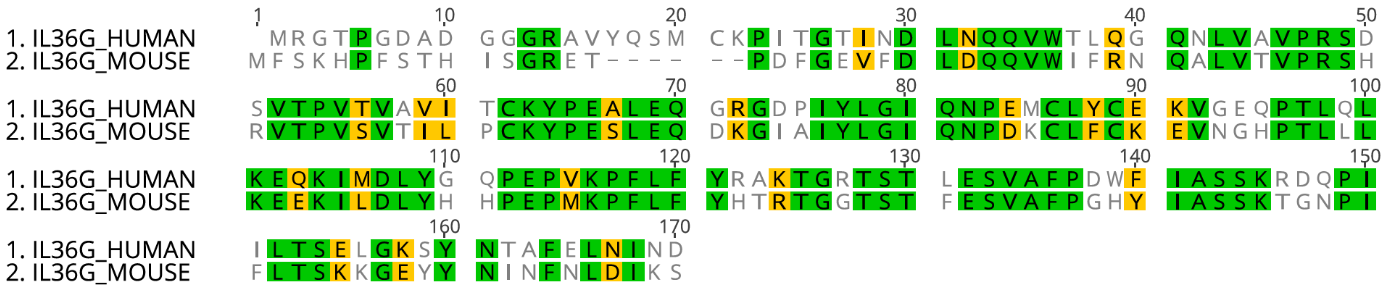

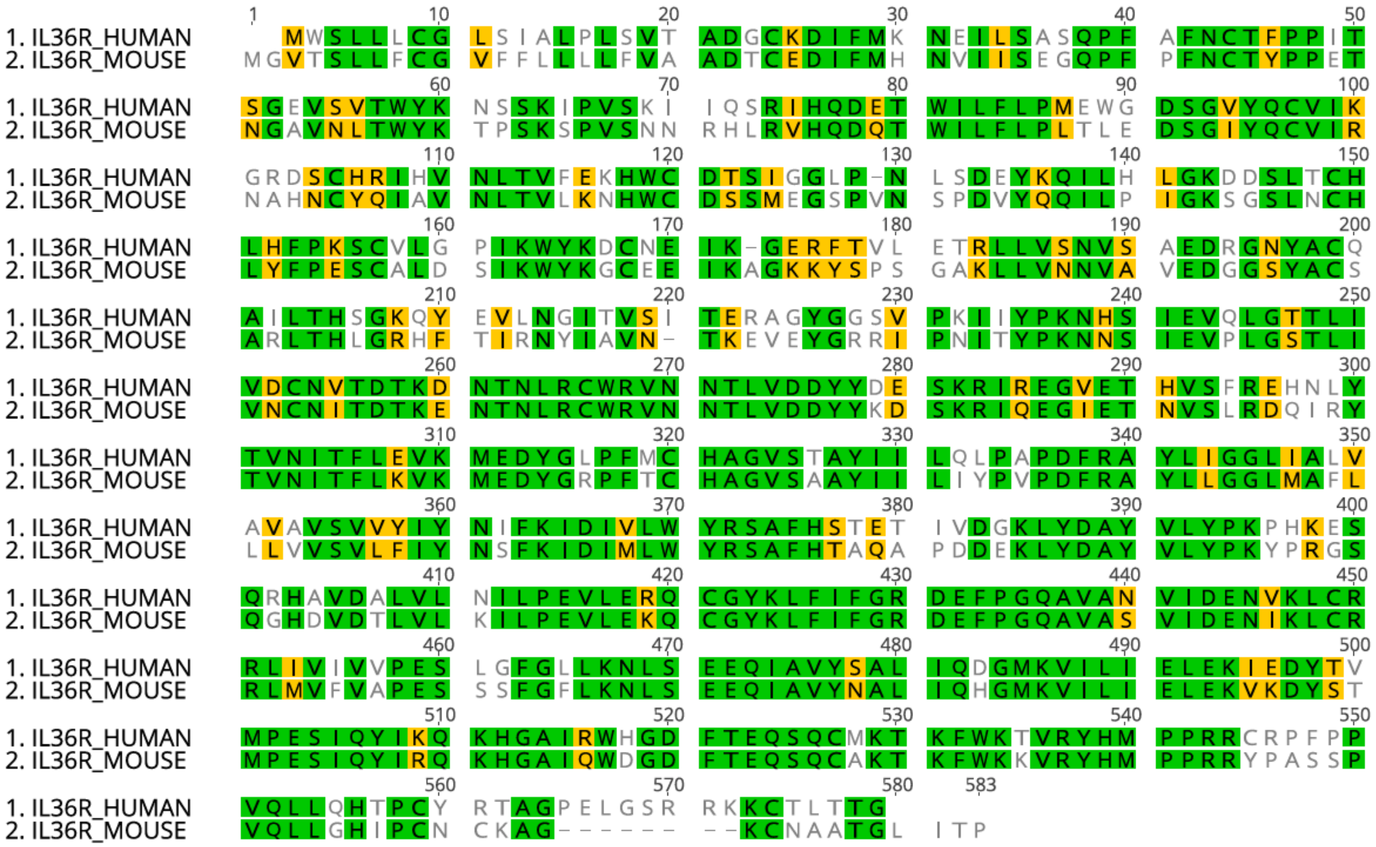

Supplementary Figure 7

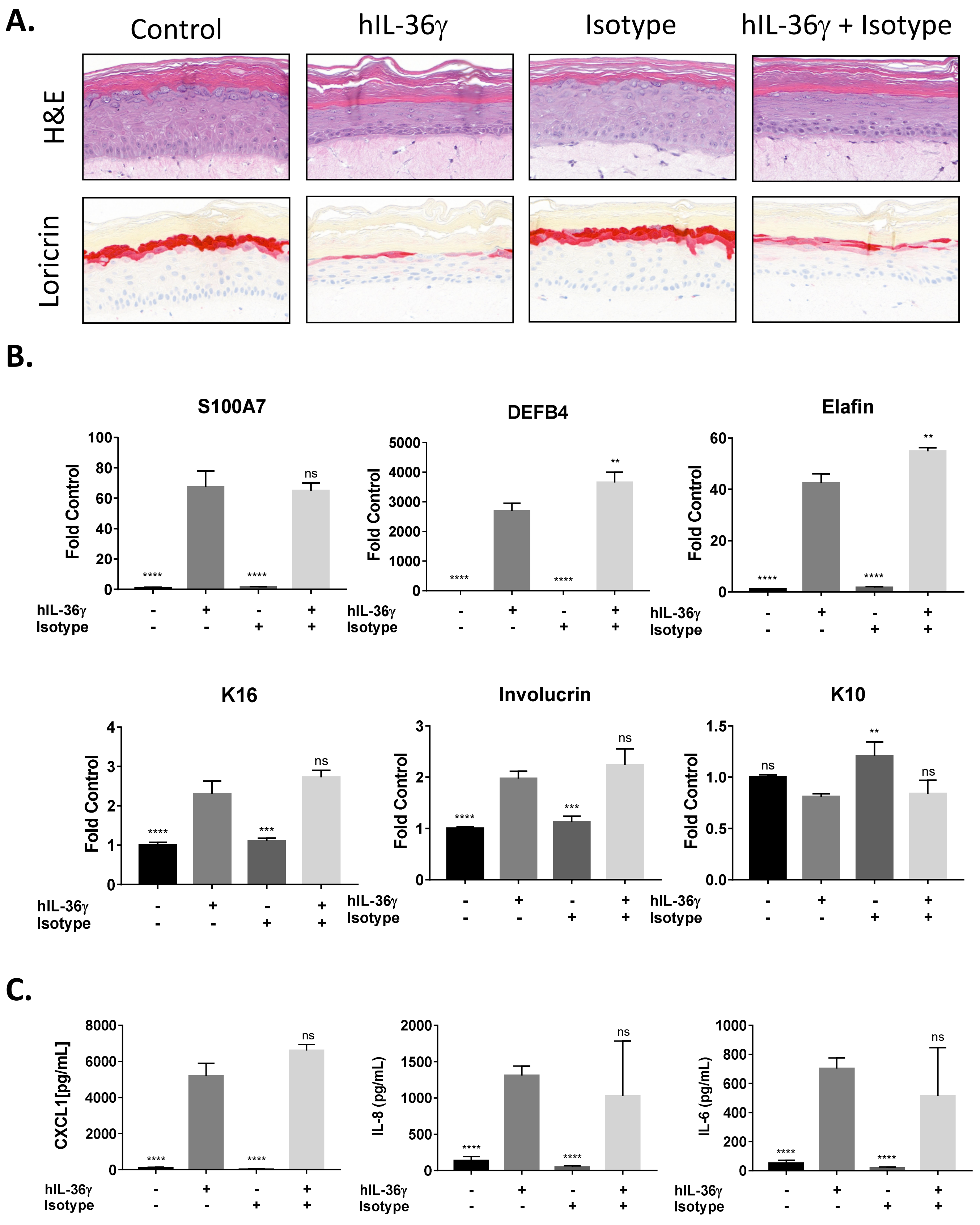

Supplementary Figure 8

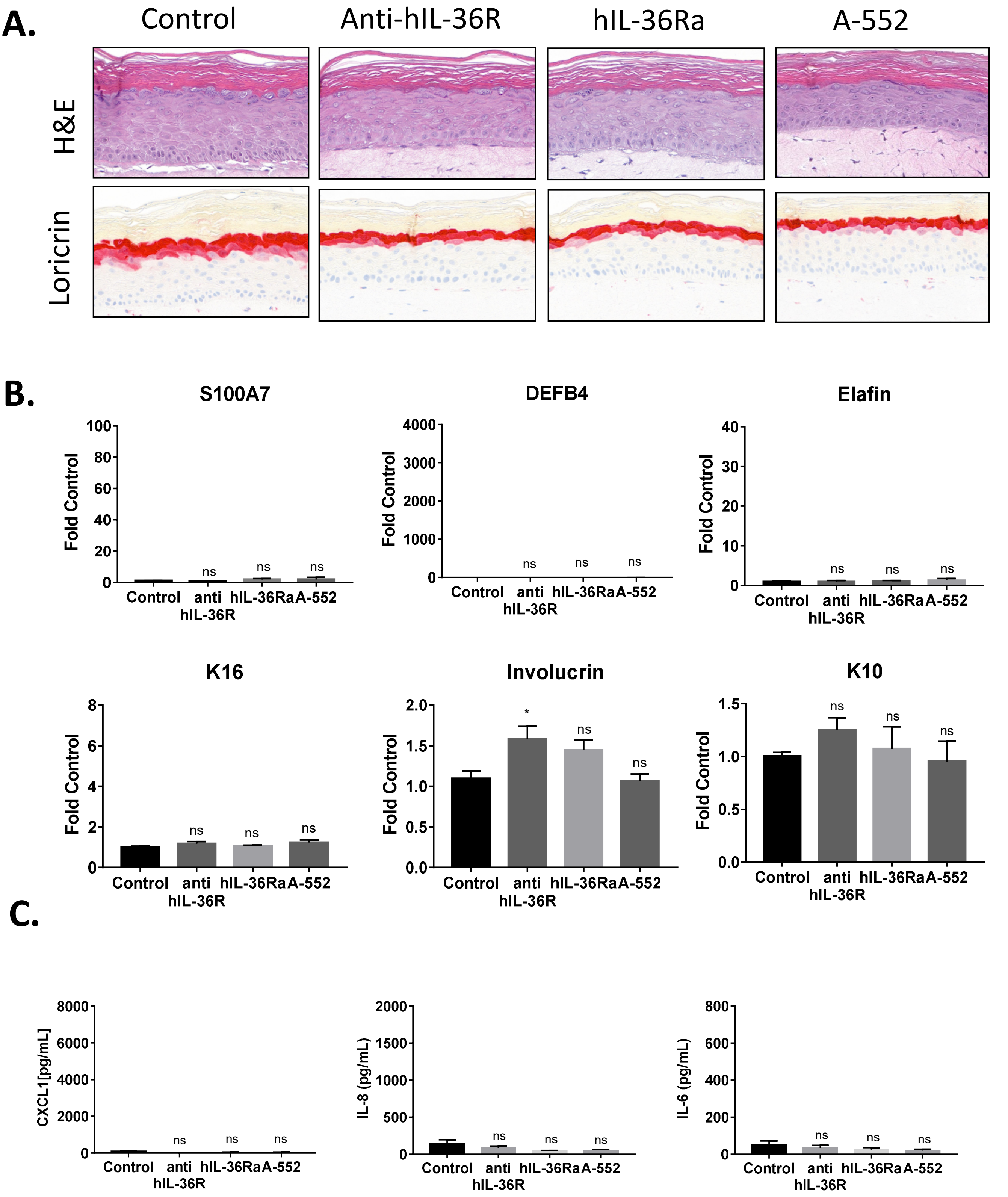

Supplement: Supplementary file 1 — Supplementary Info [file 41598_2019_45626_MOESM1_ESM.pdf]
